# Supplementary material for: The E3 ubiquitin ligase RNF115 regulates phagosome maturation and host response to bacterial infection
Source: EMBO J. 2022 Oct 25;41(23):e108970. doi: 10.15252/embj.2021108970 (PMC9713710; doi:10.15252/embj.2021108970)
Supplement: Supplementary file 1 — Appendix [file EMBJ-41-e108970-s004.docx]

**Appendix**

**The E3 ubiquitin ligase RNF115 regulates phagosome maturation and host response to bacterial infection**

**Orsolya Bilkei-Gorzo^2,3^, Tiaan Heunis^1^, José Luis Marín-Rubio^1^, Francesca Romana Cianfanelli^1^, Benjamin Bernard Armando Raymond^1^, Joseph Inns^1^, Daniela Fabrikova^2^, Julien Peltier^1,3^, Fiona Oakley^1,4^, Ralf Schmid^5,6^, Anetta Härtlova^1,2,3*^, Matthias Trost^1,3*^**

^1^Biosciences Institute, Newcastle University, Newcastle upon Tyne, UK.

^2^Wallenberg Centre for Molecular and Translational Medicine, Department of Microbiology and Immunology at Institute of Biomedicine, University of Gothenburg, Gothenburg, Sweden.

^3^MRC Protein Phosphorylation and Ubiquitylation Unit, University of Dundee, Dundee, Scotland, UK

^4^Newcastle Fibrosis Research Group, Newcastle University, Newcastle upon Tyne, UK.

^5^Leicester Institute of Structural and Chemical Biology, University of Leicester, Leicester, United Kingdom

^6^Department of Molecular and Cell Biology, University of Leicester, Leicester, United Kingdom

*Correspondence to:

Matthias Trost, Biosciences Institute, Newcastle University, Newcastle upon Tyne, UK. [matthias.trost@ncl.ac.uk](mailto:matthias.trost@ncl.ac.uk)

Anetta Härtlova, Wallenberg Centre for Molecular and Translational Medicine, University of Gothenburg, Gothenburg, Sweden. [anetta.hartlova@gu.se](mailto:anetta.hartlova@gu.se)

**Contents:**

| **Figure Number** | **Figure Title** |
| --- | --- |
| Appendix Figure S1 | Phagosomal ubiquitylation is almost entirely cytoplasmic. |
| Appendix Figure S2 | To account for people with red-green colour-blindness, we have represented Figure 1I in a magenta/cyan/yellow version. |
| Appendix Figure S3 | Ubiquitylation of vesicle trafficking proteins and TAB2-TUBE pulldown of ubiquitylated phagosomal proteins. |
| Appendix Figure S4 | Phagosomal RNF115. |
| Appendix Figure S5 | To account for people with red-green colour-blindness, we have represented Figure 5C in a magenta/cyan/yellow version. |
| Appendix Figure S6 | Loss of RNF115 does not affect phagocytosis. |
| Appendix Figure S7 | Proteomics intensity data of MAVS and STING proteins in BMDMs. |


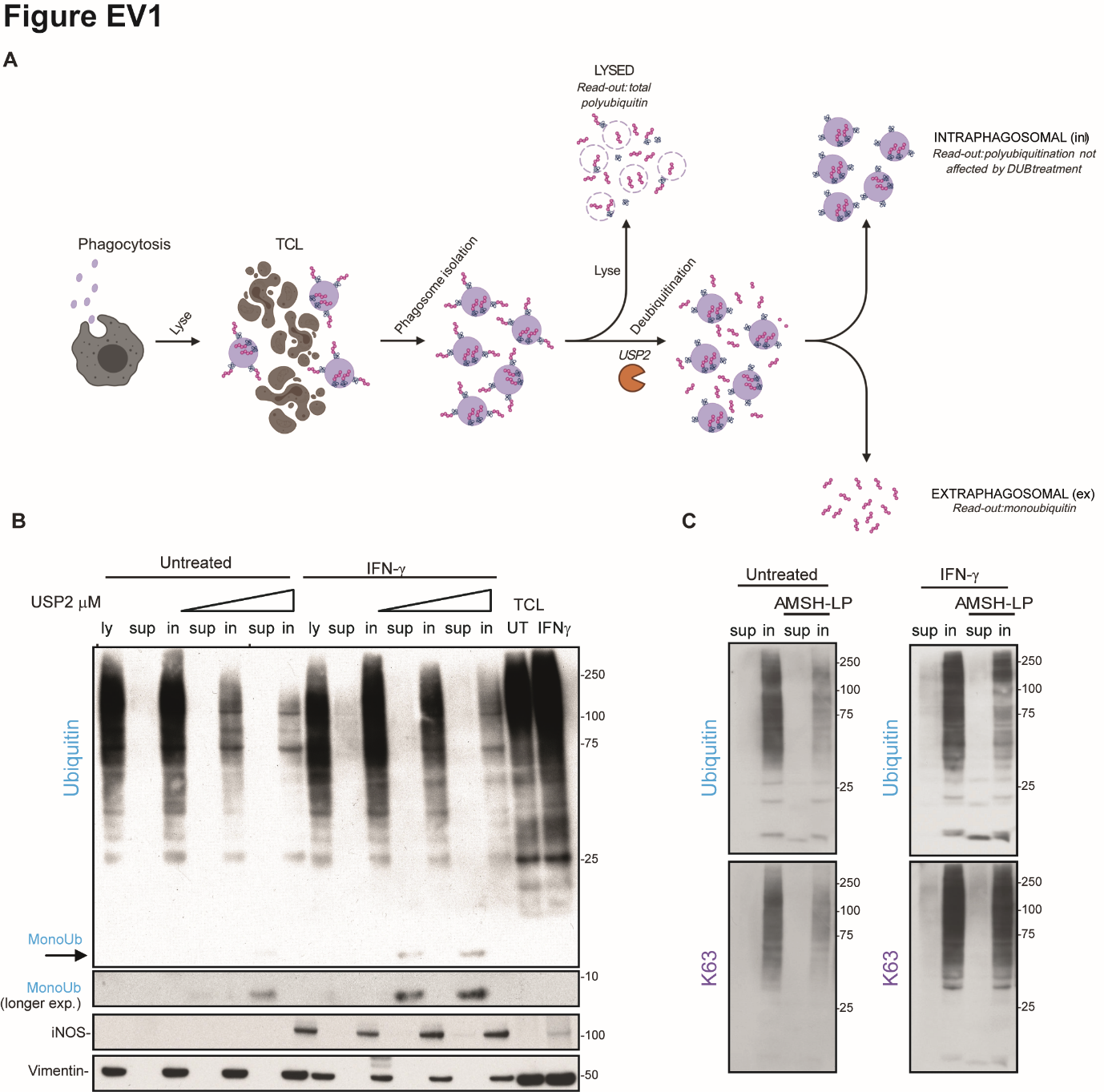


**Appendix Figure S1. Phagosomal ubiquitylation is almost entirely cytoplasmic.** (A) Workflow: phagosomes were isolated and then either lysed using a detergent containing buffer or treated with increasing concentrations of (chain unspecific) USP2 to cleave off cytoplasmic ubiquitin chains. Phagosomes were then pelleted and the supernatant or the lysed pellet was analysed by Western Blot against ubiquitin. (B) Western blot of phagosomes treated as described in A). The data show that increasing amounts of USP2 can remove ubiquitin chains from the cytoplasmic side, leaving mono ubiquitin in the supernatant (sup). Intact phagosomes (in) show reducing amounts of polyubiquitin at the same time. Lysates (ly) serve as a control. iNOS serves as a control for IFN-activation. Vimentin serves as an attempt to control loading. (C) The same treatment of phagosomes with the K63-specific AMSH-LP shows that K63 chains are highly abundant on the cytoplasmic side of phagosomes. Western blots representative of two replicates. Relative mobilities of reference proteins (masses in kDa) are shown on the right of each blot.

**
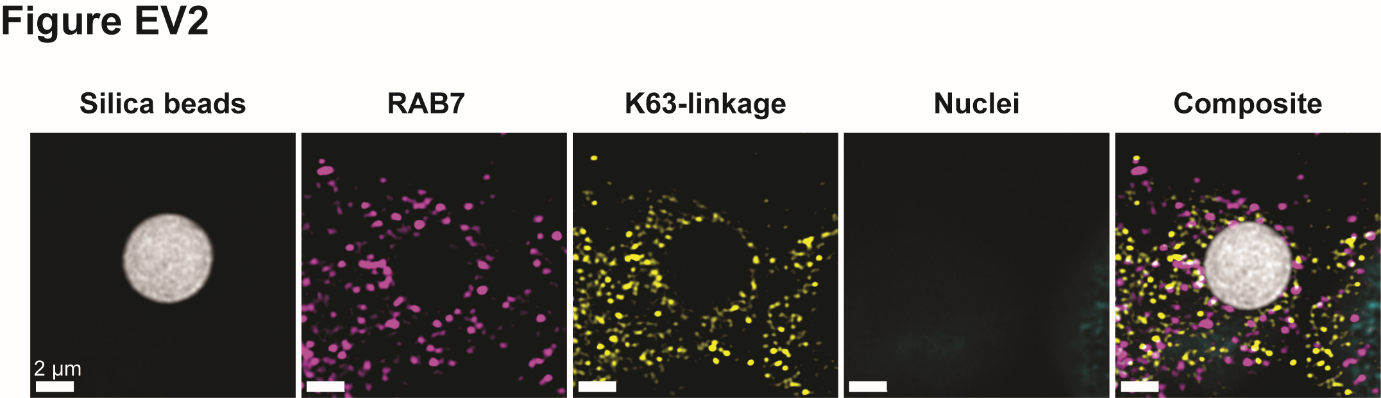
**

**Appendix Figure S2. To account for people with red-green colour-blindness, we have represented Figure 1I in a magenta/cyan/yellow version.** Representative immunofluorescence micrograph showing that K63 polyubiquitin (in yellow) localises around the phagosome defined by Rab7 staining (in magenta), and the nuclei (in cyan). Bead size (in white): 3 μm. Scale bar is 2 µm.


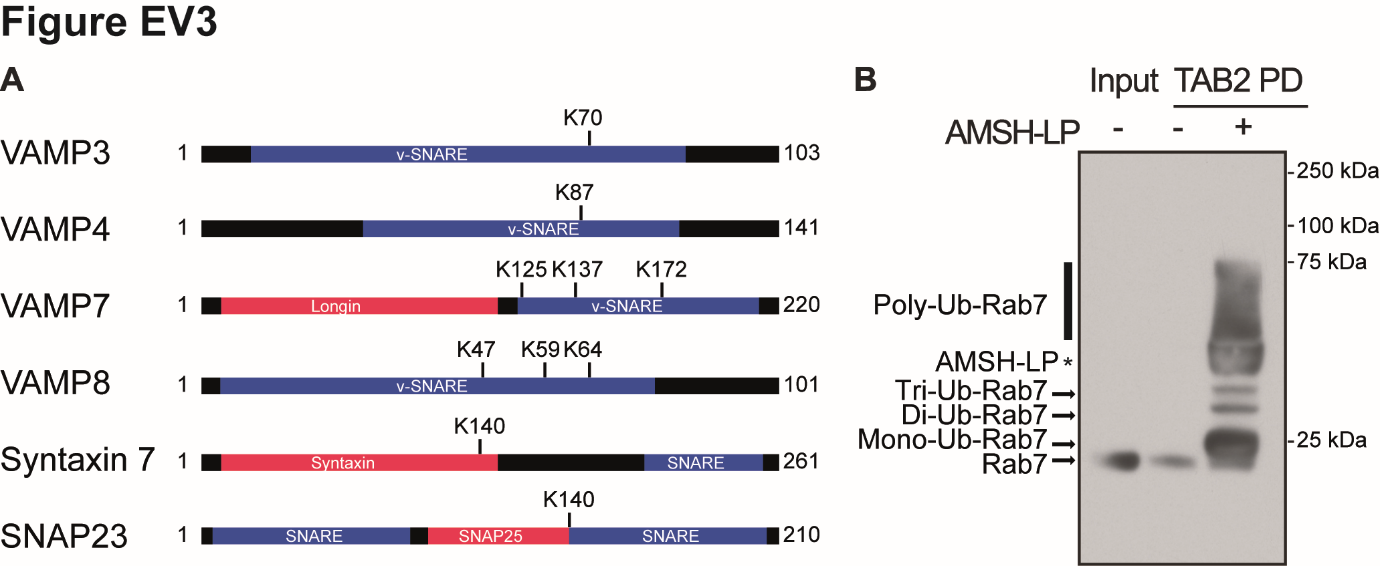


**Appendix Figure S3. Ubiquitylation of vesicle trafficking proteins and TAB2-TUBE pulldown of ubiquitylated phagosomal proteins.** (A) Ubiquitylation sites of phagosomal SNARE proteins are almost entirely within SNARE domains, thereby this might block SNARE protein interactions. (B) Ubiquitylation of proteins affects their detection by antibodies. As an example, Rab7 is shown. TAB2-NFZ TUBE pulldown (PD) of K63 polyubiquitylated proteins and subsequent Western blot of Rab7 shows no ubiquitylated forms of Rab7 (probably by blocking of antibody antigen). Upon AMSH-LP treatment multiple forms of ubiquitylated Rab7 appear. Relative mobilities of reference proteins (masses in kDa) are shown on the right of each blot.


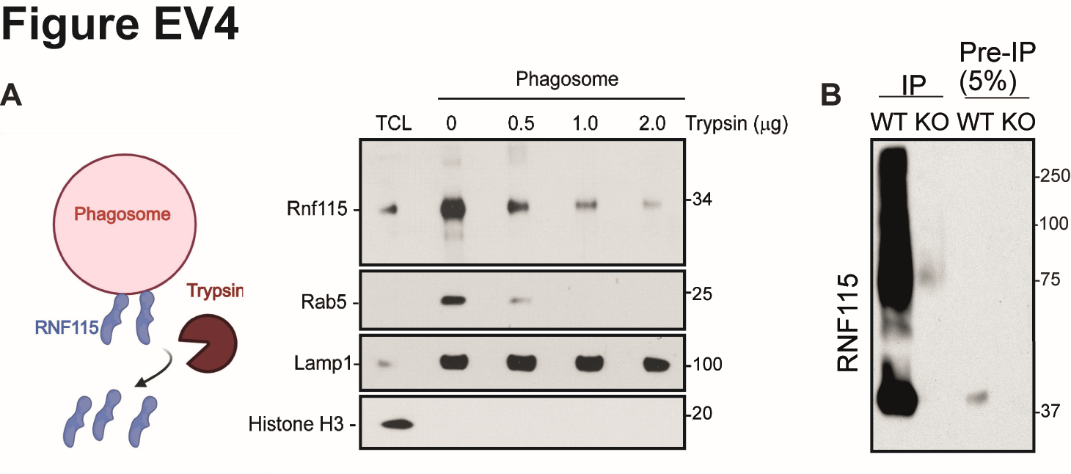


**Appendix Figure S4. Phagosomal RNF115.** (A) RNF115 is located on the cytoplasmic side of phagosomes. Treatment of isolated phagosomes with increasing amounts of Trypsin shows a reduction of RNF115. Rab5, a cytoplasmic membrane bound protein and LAMP1, a transmembrane protein, serve as controls. Histone H3 serves as purity control. Representative blot of two replicates. (B) Immunoprecipitation (IP) of RNF115 from WT and RNF115 KO macrophage cell line shows that RNF115 is absent in the RNF115 KO cells. Relative mobilities of reference proteins (masses in kDa) are shown on the right of each blot.


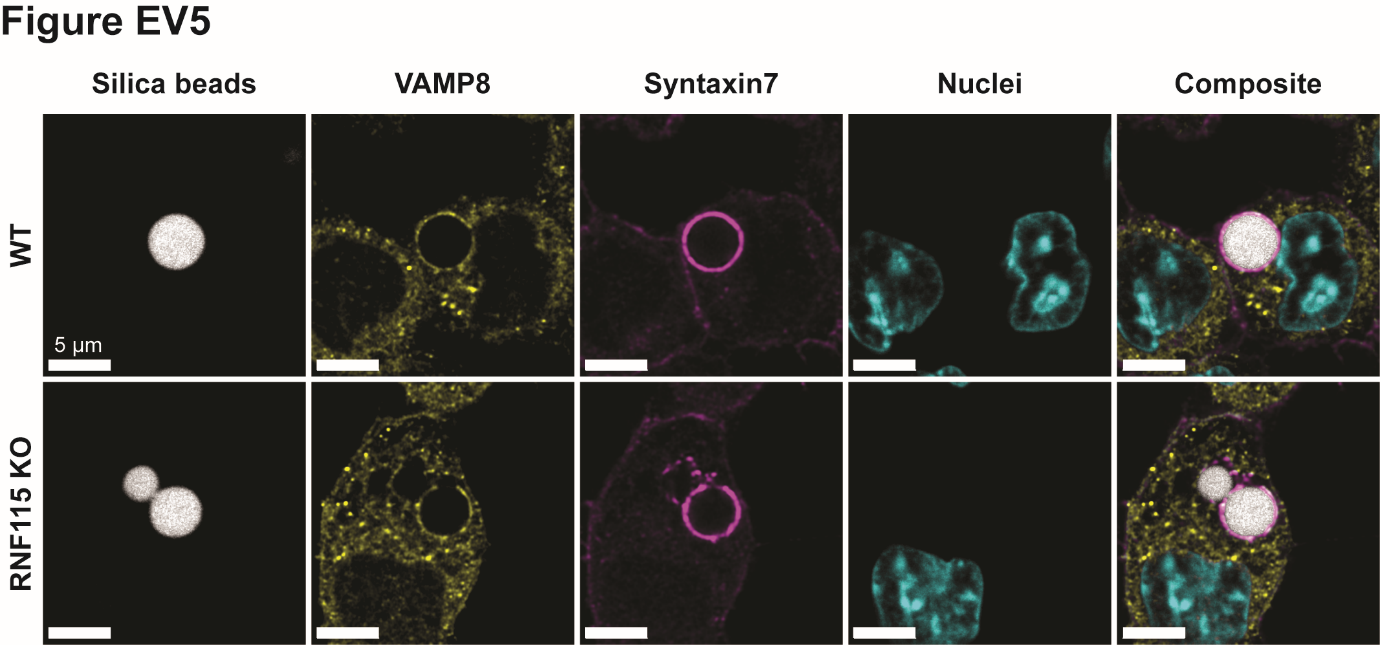


**Appendix Figure S5. To account for people with red-green colour-blindness, we have represented Figure 5C in a magenta/cyan/yellow version.** Immunofluorescence micrographs of VAMP8 (yellow) and Syntaxin-7 (STX7; magenta) show strong colocation around 2 µm silica bead phagosomes (white) in BMA macrophages. Nuclei are stained with DAPI in blue. Scale bar is 5 µm.


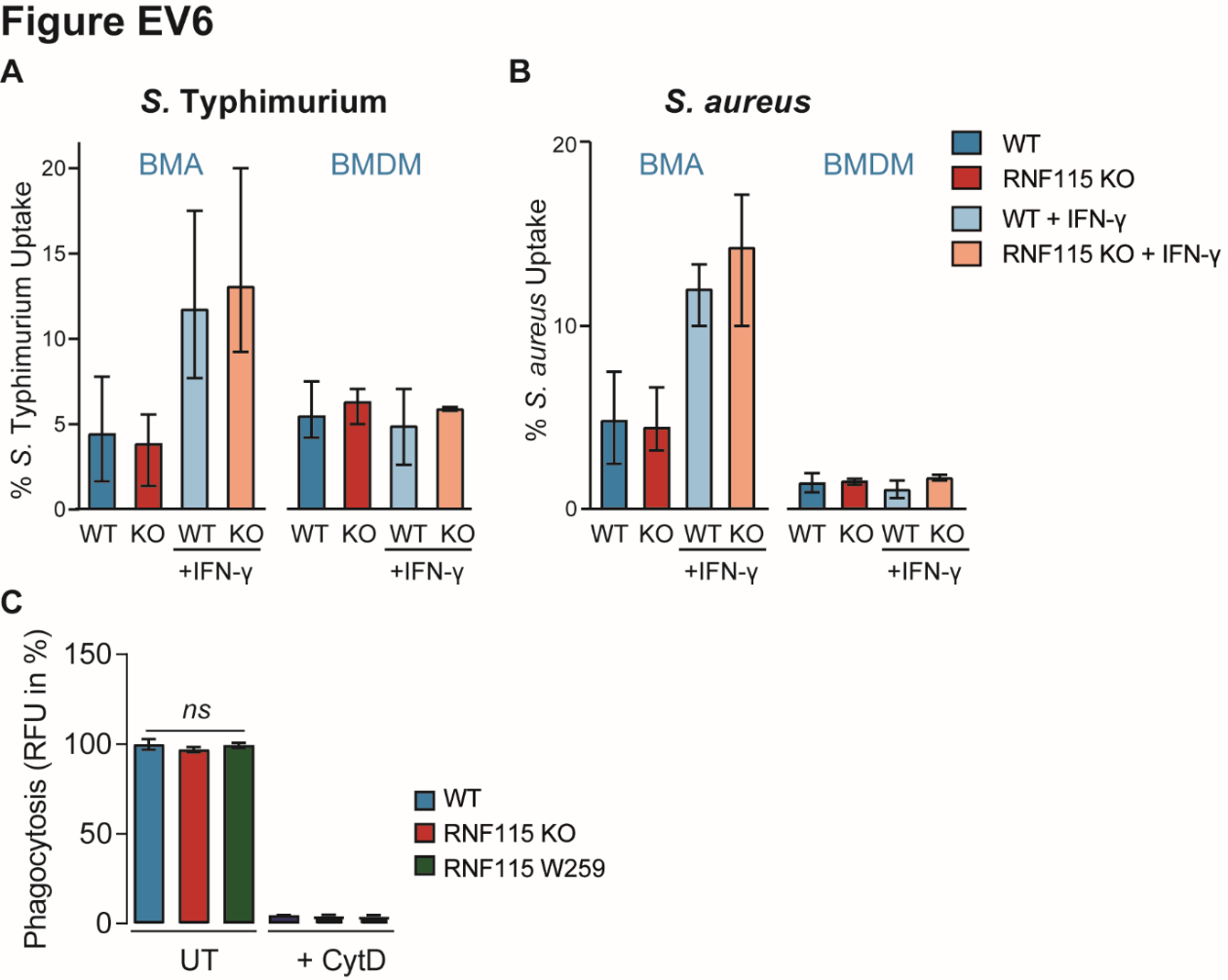


**Appendix Figure S6. Loss of RNF115 does not affect phagocytosis.** Uptake of (A) *S.* Typhimurium and (B) *S. aureus* in WT and RNF115 KO BMA cells and BMDMs is not affected by loss of RNF115. Standard deviation bars represent SD of four biological replicates. **C**) Phagocytosis of 1 μm silica beads is not affected by loss of RNF115 or expression of the “ligase-dead” RNF115 W259A in the RNF115 KO cell line. Cytochalasin D (CytD) serves as negative control as it blocks phagocytosis. Standard deviation bars represent SEM of three biological replicates.


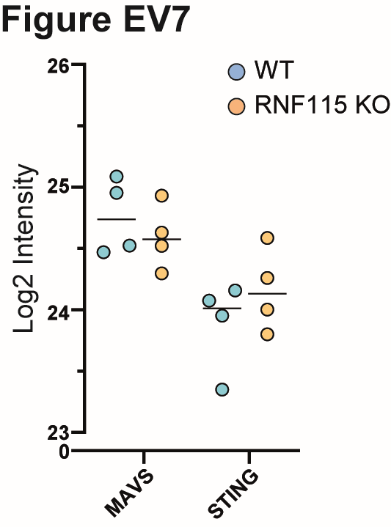


**Appendix Figure S7. Label-free protein intensity of MAVS and STING in BMDMs.** Label-free proteomics intensity data of MAVS and STING proteins in total cell lysates of BMDMs. Four biological replicates are shown.
