# Supplementary material for: The E3 ubiquitin ligase RNF115 regulates phagosome maturation and host response to bacterial infection
Source: EMBO J. 2022 Oct 25;41(23):e108970. doi: 10.15252/embj.2021108970 (PMC9713710; doi:10.15252/embj.2021108970)
Supplement: Supplementary file 6 [file EMBJ-41-e108970-s005.pdf]

THE EMBO JOURNAL

The E3 ubiquitin ligase RNF115 regulates phagosome maturation and host response to bacterial infection

Orsolya Bilkei-Gorzo, Tiaan Heunis, Jose Luis Marin-Rubio, Francesca Cianfanelli, Benjamin Raymond, Joseph Inns, Daniela Fabrikova, Julien Peltier, Fiona Oakley, Ralf Schmid, Anetta Härtlova, and Matthias Trost
DOI: 10.15252/embj.2021108970

Corresponding author(s): Matthias Trost (matthias.trost@ncl.ac.uk) , Anetta Härtlova (anetta.hartlova@gu.se)

Review Timeline:

Submission Date:	13th Jul 21
Editor correspondence:	3rd Sep 21
Author Correspondence:	16th Sep 21
Editorial Decision:	17th Sep 21
Revision Received:	25th Jul 22
Editorial Decision:	30th Aug 22
Revision Received:	1st Oct 22
Accepted:	6th Oct 22

Editor: Hartmut Vodermaier

Transaction Report:

(Note: With the exception of the correction of typographical or spelling errors that could be a source of ambiguity, letters and reports are not edited. Depending on transfer agreements, referee reports obtained elsewhere may or may not be included in this compilation. Referee reports are anonymous unless the Referee chooses to sign their reports.)

Thank you for submitting your manuscript on RNF115 in phagosome maturation and antibacterial response to The EMBO Journal. I apologize for the delay in getting back to you with a decision, owed to limited referee availability and the need to grant extended reviewing deadlines during the summer vacation season. We have now received the below-copied reports from four reviewers covering the different expertise areas relevant to the study. As you will see, the referees acknowledge the importance of the topic and the potential interest of your findings. At the same time, they however raise a number of overlapping concerns particularly regarding the depth of the obtained understanding and the decisiveness of the mechanistic conclusions of the study.

For publication in The EMBO Journal, it would in my view be important to not only solidify the present core findings wherever the referees felt necessary; but to also deepen the mechanistic insight to a certain degree, e.g. by determining the importance of RNF115 catalytic activity in the process, and possibly either learning more about its substrates or about how its loss affects phagosome maturation (e.g. via protein recruitment, phagosome-lysosomal fusion, etc.). While I appreciate that it could not be expected to address every individual major point of the referees experimentally, I feel that it would in this case be helpful to give you an opportunity to carefully consider all points raised together with your collaborators, and to provide a tentative point-by-point response proposing how might answer the reviewers' criticisms and extend the study should you be given the opportunity to revise this work for The EMBO Journal. With your proposal in hand, we could then schedule a Zoom call to directly discuss what could and what could not be done here, before taking a definitive decision on this manuscript. It would be great if you could get back to me with such a response over the course of the next week.

Referee #1 (Report for Author)

The manuscript EMBOJ-2021-108970 by Bilkei-Gorzo et al. describes the proteomic analysis of phagosomes in resident and in IFNg stimulated macrophage-like cells. Whereas the authors' previous work was more concentrated on the general composition of phagosomes in resident and activated cells, they now focus on quality and quantity of protein ubiquitylation on phagosomes under either condition. Using sophisticated mass spectrometry and immunoblotting, they describe for the first time the diversity of ubiquitin chain links on phagosomes vs complete cell lysates and they identify five E3 ubiquitin ligases that are present at increased concentrations after macrophage activation. One of them, RNF115, was studied in more detail and the authors provide evidence that this ligase promotes phagolysosome formation in activated cells and that it participates in the down-regulation of pro-inflammatory cytokines during infection. Therefore, this study is the first to connect a specific E3 ligase to phagosome immune effector functions. Overall, this is a well-done study

which furthers the molecular understanding of phagosome maturation which will stimulate many scientists to consider postranslational protein modifications in general and ubiquitylation in particular as important regulators of phagosome development. A number of points need to be addressed:

Major comments

1. The Supplemental Tables are not self-explanatory. In fact they did not come with any text. Please include instructive table legends for the uninitiated readers in the supplemental information section.

2. The authors use vimentin as a loading control (e.g., Fig 1C-E). However, vimentin concentrations on phagosomes change with maturation as do, understandably, the concentrations of EEA1 (Fig. 1D). How can it then be used as loading control (l. 154)? Then, in Fig S1B, vimentin is used as a control protein indicating that luminal proteins are not digested during treatment with the deubiquitinase USP2. I understand that the fact that there are normal vimentin concentrations in the treated sample is interpreted as USP2 did not act as a general protease. But why then is vimentin, a cytosolic cytoskeletal protein, present in the lumen of phagosomes ("in" in Fig S1B)? And, thirdly, why then does "the presence of vimentin on the phagosomal membrane demonstrate(d) that phagosomes remained intact", i.e., show that they are not permeabilized (l. 139-140)?

3. With reference to the infection experiments shown in Fig. 4E: This is a flow-cytometry experiment but no "Materials and Methods" were given. One big disadvantage of a flow cytometry experiment for intracellular bacterial killing is that bacteria attached to macrophages will yield a signal even when killed by antibiotics. Second, it would have been helpful not to only see the 6 h incubation result but also the initial ("0 h") starting live bacterial cell number which would help to evaluate whether multiplication and/or killing occurred (differentially). Third, the datasets for the same experiment but from IFNg-activated BMA cells is needed as this manuscript centers around an E3 ligase that is more concentrated on phagosomes in activated vs resident macrophages. Forth, a much more established experiment to quantify live intracellular bacteria at times 0 and 6 h of infection is the 'gentamicin protection assay'. Actually, the authors have described this technique in their Materials-and-Methods section (l. 645-653) but there is no corresponding data set in the manuscript. This would be the perfect experiment to use using samples with and without IFNg activation at 0 and 6 h of infection. Fifth, the authors should make clearer that Listeria is not the paradigm cytosolic pathogen but that other intracellular pathogens (Shigella, Francisella, Rickettsia and more) also break their phagosome membranes at various times of infection which may expose them much longer than Listeria to the action of RNF115 before they actually break out. Vice versa, S. aureus is taken by the authors as a paradigm for phagosomal pathogens but like all intra-host cell phagosomes/vacuoles the S. aureus phagosome is unique in its composition and certainly not representative for all bacteria/pathogens. Conclusions are to be made cautiously and not generally ("vacuolar" vs "cytosolic" pathogen). It would have been more revealing if a non-replicating, intra-phagosomal bacterium would have been used instead of S. aureus, such as apathogenic Listeria innocua which shares many features with L. monocytogenes but lacks all 'real' virulence factors.

4. Re. the experiment in which the authors show that ubiquitination can dramatically affect the recognition of a cellular protein by an antibody (a very interesting side aspect of the paper): L. 211 states "as this is the area against which antibodies were raised". It is likely "against which our antibodies were raised" making this a more special than a general observation? But, more importantly it should be mentioned that the antibodies used here were raised against a synthetic peptide which therefore could not be modified vs an authentic mammalian protein which could be ubiquitinylated. Still, how the conclusions were obtained, remains unclear because: (1) The

ubiquitination sites on Rab7 in vivo are K38, K191 and K194 (Modica and Lefrancois, Small GTPases 11: 167) and (2) the monoclonal antibody from Cell Signaling Technologies which was used here is raised to a "peptide corresponding to residues surrounding Glu188 of human Rab7 protein." So one must assume that the residues K191 and K194 were relevant here and that the antigenic peptide only become visible after deubiquitination. But then there would be no higher-molecular weight ubiquitinated Rab7 forms to be detected by the antibody: Ubiquitin there: No antibody binding - ubiquitin gone: Antibody binding but no higher molecular weight forms. Please explain!

5. The effect of RNF115 knockout on phagosome acidification (l. 297; Fig. 4B) is minor and its evaluation therefore depends even more than usually on the statistics approach used. Here, "error bars were generated from standard error of the mean of 6 replicates". Please indicate whether these were biological replicates (independent experiments) or whether these were technical replicates (parallel samples) which would not be adequate (Vaux et al., EMBO Reports, 2012). Also consider the fact that the zero time data of the RNF115 KO cells is already somewhat higher than for WT.
On a similar note, the experiment in Fig 1 F has been performed 5 times with 3 technical replicates per treatment. The authors state that due to the inherent variability of this complex experiment they combined all of the data acquired, yielding 15 (5 x 3 technical replicates) data points per condition. However, the way use these data, each technical replicate is considered an independent experiment. This is misleading, when it comes to determination of the standard error of the mean (SEM): SEM depends on sample size n (n, biological replicates) which here is actually 5, not 15. Since the authors show all of the data points, the variability becomes obvious even without SEM. Moreover, as t test p-values depend on SEM and sample size n, increasing the sample size to 15 may erroneously indicate that the differences observed are statistically significant. The authors should rather calculate means of the technical replicates for each of the independent experiments and show these instead (5 data points = means per condition), as in Fig 1 G.

7. l. 97: "Total ubiquitylation stays almost constant over the whole phagosomal maturation process of both resting and IFNg-treated macrophages (Figure 1 D)." Fig 1 D only shows the kinetics of acquisition of ubiquitylated proteins by phagosomes in resting cells.

Minor comments
8. l. 83, "delays" instead of "reduces"

9. l. 91, "pure" instead of "highly pure", l. 144: "abundant" instead of "highly abundant", l. 200 "ubiquitylated" instead of "highly ubiquitylated", particularly as it is hard to see how the level of ubiquitination was determined by the techniques used here.

10. l. 113-114, Figs 1F and 1G mixed up?

11. l. 261: CRISPR/Cas9 editing of RAW264.7 cells: Not mentioned in Materials and Methods, only for BMA cell line.

12. l. 264: "To determine whether RNF115 is involved in the regulation of phagocytosis": Was there reason to believe it would be?

13. l. 399: Please explain "ISGylation"

14. l. 410 and 1066: Is there precedence of SNARE protein motifs being inactivated by SNARE motif

ubiquitylation or is this just speculation?

15. l. 499: The description of the recombinant proteins used in this study is extremely brief. Are all or any of these proteins mouse enzymes? It would be helpful to have database accession numbers for the sequence/identity or literature reference(s). Which was the E. coli strain used?

16. l. 576: Most other experiments were made using RAW264.7 cells. Please explain why an alternative cell line was used here.

17. Mat & Meth: What kind of cells are the RAW264.7 and BMA3.1A7 cell lines used here? Please explain in the main text.

18. Fig 4G, the ELISA experiment needs to be described in more detail, both in the figure legend and the material and methods section (experimental details, sample sizes, biological versus technical replicates).

19. l. 103, the ubiquitin-chain type specific antibodies are not specified in main text, figure legend, or Material and Methods section. Further information would be appreciated.

20. Some E3 ligases are enriched in phagosomes from IFNg-treated cells over phagosomes from untreated cells. However, in Fig 2 C it reads "IFNg only" for these E3 ligases, suggesting that these E3 ligases were absent from resting cell phagosomes. What is the threshold for the authors to define an E3 ligase as enriched?

21. The introduction should include a few sentences about IFNg and its relevant effects on macrophages and phagosome biogenesis.

22. Fig 1C/D: The authors should comment in the main text or figure legend how phagosome and TCL sample concentrations were adjusted to each other. This is especially important for the conclusion that ubiquitylated proteins are enriched on phagosomes as compared to the TCL.

23. Fig 1 F: 'M1' is not specified, in Figure 1 H 'UT' is not specified

24. Fig 1I: A scale bar is missing. Moreover, a brightfield image would be appreciated to show the position of the phagocytosed silica bead. A brightfield image should be included in Fig 3 C, too.

25. Fig 2F legend reads: "Level of shading of edges indicates interaction confidence." It rather seems as if the shading of the connecting lines indicates interaction confidence.

26. Axis titles should describe more directly what the authors have measured. This applies especially to Fig 5.

Additional non-essential suggestions for improving the study

27. The authors show here that they have the technology to identify ubiquitinylated proteins and that they have identified an E3 ligase which is required to exert immune functions during activation. It would be very interesting to see which phagosomal proteins precisely are ubiquitinylated by RNF115 in activated versus non-activated macrophages and in RNF115 WT versus KO cells, particularly as this may identify some obvious and interesting further candidates to follow up.

Referee #2 (Report for Author)

In the manuscript by Mathias Trost and colleagues, the authors investigated the role of RNF115, a ubiquitin ligase of the RING Finger family, in regulating phagosomal function. The authors show that RNF15 acts as a classical E3 ubiquitin ligase that it affects ubiquitylation of phagosome associated proteins and subsequently regulates innate responses to S.aureus in vitro and in vivo.

Strengths of the manuscript

The discovery of RNF115 to be an E3 ligase involved in modulating phagosome maturation is novel. In particular, it seems to delay dynamics of maturation. ubiquitylation of vesicle transport related proteins, some of which might be direct substrates.
RNF115 mediated ubiquitylation directly affects S.Aureus infection dynamics, demonstrating a role of ubiquitylation in the associated innate immune response.
These are all new findings are are worth publishing. In addition, the manuscript is well written, and the experiments have been carried out and are described with a high technical standard.

Weaknesses of the manuscript

Figure 2 shows the GlyGly modified proteins described in this study. The authors show that also components of the UBL system, in particular ISG15, appears to be enriched upon IFNgamma induction in their cellular system. As Ub and ISG15 share the same C-terminal sequence, ISG15ylated and Ubiquitylated proteins, upon trypsin digestion, result both in GlyGly-modified peptide fragments.

Therefore, there should be some caution in assigning all GlyGly-K sites identified to modifications by ubiquitin (e.g. Figure 2F). Granted, ISGylation is
predominantly induced by IFNalpha/beta, but this might be intertwined as the authors show with increased levels of Herc6 (Figure A), an E3 ligase that might conjugate ISG15 rather than ubiquitin (not completely established yet). Therefore, it is recommended that the authors should carefully compare their GlyGly proteomics data to know ISG15ylation signatures, now readily available in the literature.

K11 ubiquitylation seems the linkage type mostly accumulating upon IFNgamma exposure. This appears to be an interesting observation. The authors do not really follow up on their Ub-linkage characterisation (Figure 1) in the experiments shown in the rest of the manuscript. Also, the authors state the effect of RNF115 on the ubiquitylation status of a panel of phagosome associated proteins without going into direct enzyme-substrate relationships. However, the latter may require substantial additional work and maybe outside the scope of this work.

There appears to be a discrepancy between RNF115 delaying phagosome maturation, but at the same time, enhancing immune responses that involved phagosome mediated microbial uptake. Also, reduced inflammation/tissue damage upon RNF115 removal, as shown by the authors in Figure 5, does not seem to be consistent with enhanced capacity to clear microbial threats. Could

the authors further elaborate on how RNF115 fine-tunes phagosome ubiquitylation to achieve this?

In summary, this is a novel and interesting study that, upon addressing the concerns listed above in a convincing manner, should be considered for potential publication.

Referee #3 (Report for Author)

- General summary and opinion about the principal significance of the study, its questions and findings:

In this study, Bilkeu-Gorzo et al. display a sequence of 3 quantitative proteomic analyses to investigate the regulation of the phagosome ubiquitinome. First, they compare the ubiquitylation of isolated phagosomes with the total cell lysate (TCL) in the presence or absence of IFN-gamma and demonstrate increased ubiquitylation of phagosomes upon IFN-gamma activation. Besides, they identify the ubiquitin chains highly present in phagosomes compare to TCL and increased upon IFN-gamma activation. Second, they analyze the proteome and ubiquitinome of isolated-phagosomes. They showed an increased abundance of E1, E2, and E3 ligases at the phagosome upon IFN-gamma activation, as well as increased ubiquitylation of innate immunity and vesicle trafficking proteins. Focusing on one of the E3 ligases, RNF115, they perform a third proteomic analysis comparing the phagosome proteome of WT and RNF115 KO cells and show that loss of RNF115 affects the abundance of many proteins at the phagosome. They do not investigate further these proteins, nor confirmed those hits. Finally, they observe that loss of RNF115 affects phagosome proteolysis and acidification, as well as immune response during bacterial infection in macrophages and in vivo. The mechanism is not addressed.

The quantity of proteomic information gathered in this study is consequent and contains a true potential for a better understanding of phagosome biology, which is the strong point of the study. Yet, while many proteins are identified, the data are not integrated to reach a mechanistic understanding of the role of (IFN-gamma-induced) ubiquitylation of the phagosome. Many additional experiments should be implemented to test the hypothesis opened by the proteomic approaches. In the manuscript's current state, the mechanistic conclusions are mostly overinterpreted /not supported by the data.

- Specific major concerns essential to be addressed to support the conclusions:

1_ Additional experiments should be performed to provide a mechanistic understanding of the role of IFN-gamma-induced ubiquitylation of the phagosome. Notably, the link between IFN-gamma activation and the function of RNF115 in phagosomal ubiquitylation and maturation should be established (for instance, +/- IFN-gamma conditions may be included from Fig 4b onward and the ubiquitin chain affected by RNF115 in the presence/absence of IFN-gamma may be tested). In particular, the results obtained with RNF115 KO should be tested with the RNF115 W259A mutant in order to conclude on the role of RNF115 ubiquitylation activity. To capitalize on the results obtained by the third proteomic analysis (RNF115 KO vs WT), the authors should confirm some of the most promising hits identified and test their recruitment to the phagosome under RNF115 W259A expression. Finally, the study would highly beneficiate from linking one of the proteins (or

complexes) significantly depleted or enriched at the phagosome upon RNF115 KO with the RNF115-controlled phagosome maturation.

2_Regarding the infection experiments, the experimental setting does not support the conclusion that RNF115 KO increases degradation of phagosomal S. aureus but not cytosolic Listeria because the bacterial localizations are not tested. Indeed, the localization of S. aureus and L. monocytogenes are predicted from the literature but not shown by the data. It is now widely established that despite the main cytosolic lifestyle of L. monocytogenes, a subpopulation of infected cells contains vacuolar Listeria. Besides, as mentioned by the authors, S. aureus is mainly extracellular and only a small number of them are internalized into phagosomes. As the authors extrapolate on the link between RNF115 effect and the pathogen intracellular localization ("This suggests that loss of RNF115 affects specifically innate immune signalling from the phagosome, while signalling from the cytosol is unaffected." Lines 340-342), the experimental design should minimize all differences in the pathogenicity other than bacteria localization (avoiding divergent effects of species-specific bacterial effectors). An alternative would be to use bacteria that can display both cytosolic and vacuolar lifestyles such as Salmonella. Here a fluorescent reporter of the bacteria localization would be particularly suited for the authors' flow cytometry approaches. The cytosolic and vacuolar subpopulations can be easily enhanced using mutant strains.

3_ Generating RNF115 KO mice is a great asset for in vivo investigations of the role of RNF115 during bacterial infections. Yet, this tool is barely exploited in the manuscript and does not support the conclusion that RNF115 enhances bacterial clearance. In particular, the authors should quantify the number of bacteria and not restrict their analysis to the liver only. In addition, performing bacteria subcutaneous/local injection rather than intravenous injection may be more appropriate to quantify tissue damage.

4_ The current version of the manuscript contains numerous mistakes in the text and figures. Individually taken, these concerns are minor, but due to their high number, they significantly impair the understanding of the manuscript and the trust in the data quality. The authors should make a thorough check of the figures, main text, and captions to correct and clarify any similar problem in addition to the one listed in the section below.

- Minor concerns that should be addressed (listed in order of appearance in the manuscript):

- Fig 1D: the text says "both resting and IFN-gamma treated macrophages" but only one condition is displayed and no information regarding the IFN-gamma treatment appears in the caption.
- Fig 1D: Why is the vimentin band unequal between conditions?
- Fig 1E: EEA1 should be included as a purity marker like in Fig 1C-D.
- The text should include a note on the fact that IFN-gamma stimulation does not significantly affect phagocytosis (ie, the number of phagosomes formed is similar with/without IFN-gamma).
- Fig 1H: "UT" should be explained in the caption or text.
- The introduction of the different DUBs specificity lines 116-119 and Fig 1H lacks clarity and is sometimes inconsistent between text and figure. The meaning of the signs ">" and ">>" should be clearly stated. A suggestion would be to replace the top left box of Fig 1H with a table crossing the mentioned DUBs and their targets.
- Fig 1.I : A panel showing the full cell (like in Fig 3C) should be included.
- Fig 1.I: The scale bar is missing.
- Fig. 1I: Why beads of 3 microns of diameters are used here while the size used for phagosome isolation is 0.8 micron?

- The caption of Fig 1C-E should be untangled. It is currently hard to follow.
- In the paragraphs contained between lines 168-190, most references to Fig 2 subpanels are incorrect.
- Fig 2F is not described in the text.
- Supplementary Fig 2B is not described in the text.
- In Supplementary Fig 2, what do the blue and red parts of the SNARE sequences correspond to? This should appear in the caption. Besides TAB2 PD should be introduced in the caption (ie, "TAB2 PD is a K63-chain binding protein").
- Lines 220, 223, and Fig 3E, the term "upregulated" is incorrect and should be replaced by "enriched" as it refers to protein abundance on a subcellular compartment.
- Fig 3A: The results displayed are confusing in regard to the previous figures. My understanding is that the E3 ligases plotted in Fig 3A are selected from the list plotted in Fig 2C (this information, if correct, should appear somewhere in the text or caption, as well as the selection criteria). However, why Herc6 and RNF115 are written "IFN only" in Fig 2C - suggesting that they were below the detection level without IFN stimulation - but their log2 intensity in the unstimulated condition is display in Fig 3A?
- Fig 3A: The carried t-tests show 4 stars in the comparison of RNF115 enrichment between Ctrl and IFN-gamma treatment but only 2 stars for Herc6 (which by eye look similar). To dissipate any misunderstanding, the authors may provide the raw data used for this figure in their rebuttal.
- Fig 3B: Rab7 cannot be used as a purity control as it interacts with RNF115 (as explained in lines 228-231) and should be removed from the plot.
- Fig 3C: a comparison with RNF115 recruitment at the phagosome without IFN activation would be relevant, and quantification would make the results more convincing.
- Fig 3D: The proteins named within the volcano plot are not mentioned in the text or caption. It would be preferable to point at proteins that are later mentioned in the text (such as the one present in Fig 3F).
- The results displayed in Supplementary Fig 3C-D are described lines 263-268 as such: "Ablation of RNF115 or loss of E2 binding of RNF115 did not affect phagocytic uptake of carboxylated particles (supplementary figure 3C) or bacterial uptake (supplementary figure 3D)." Yet, RNF115 mutant is only introduced in line 301, and the bacteria in line 327. I believe that the authors may have wrongly reshuffled the position of this line in their manuscript. This needs to be re-organized (see also additional suggestions).
- In Supplementary Fig 3: Does "RFU" stands for "relative fluorescence units" and "CytD" for "CytochalasinD"? This should appear in the caption. Similarly, the reason why CytochalasinD is used (I guess as a negative control ?) should be stated somewhere. The material and method section describes CFU determination but this does not appear in any figure. Maybe the authors meant "CFU" instead of "RFU" in the supplementary Fig 3D?
- Supplementary Fig 3D: The condition "RNF115 W259A" should be included.
- Lines 278-279: the statement "loss of RNF115 also decreased recruitment of the ESCRT complex highlighting the role in ubiquitylation for the functional role of the complex" is not supported by the data because RNF115 is depleted but not replace by RNF115 W259A. Additional experiments should be performed or the statement should be corrected. Same comment for line 290 " this data shows that ubiquitylation plays an important role in protein and vesicle trafficking."
- Line 283, the authors wrote "TRAPP was significantly less abundant on RNF115 KO phagosomes and may explain the retention of cargo that was meant to be secreted". Was this retention of cargo observed? If yes, the authors should include such data or references to published work.
- Fig 4A: the result of IFN-gamma treatment is not commented in the text.
- Fig 5B: what is Bafilomycin used for? This and the interpretation of the results should appear in the text (or be removed from the figure).

- Fig 5B: The conditions +/-IFN gamma treatment should be included.
- Fig 5B: Strange lines appear around "WT+Bafi" and "RNF115+Bafi" lines instead of shaded area representing SEM.
- Lines 298-300: " These data were further validated by complementing RNF115 KO with RNF115-HA that reversed enhanced phagosomal proteolytic activity of KO macrophages". Where are the data?
- Lines 335-336: "The analysis revealed that RNF115 KO deficiency did not impact uptake of either pathogen" refers to results displayed in Supplementary Fig 4D, not Fig 4E. The following sentence "However, degradation of phagosomal S. aureus was significantly increased in the KO, while cytosolic Listeria were not affected" most likely refers to Fig 4E.
- Fig 4E: the "non-infected" condition must be shown to determine the gate of GFP-positive signal versus autofluorescence.
- Fig 4E: the data doesn't allow to decipher if the lack of intracellular S. aureus is due to bacterial clearance or lack of replication. Several time points should be displayed to provide this information.
- Lines 366-367: the link between CD68-stained macrophage and tissue repair should be justified by literature references.
- Line 368: the conclusion on bacterial clearance is not supported by the data as the bacteria have not been quantified.
- Fig 5: Adding a condition with/without IFN-gamma stimulation would strengthen the results.
- In the material and methods, the bacteria are not described as fluorescent, but S. aureus GFP and L. monocytogenes eGFP are used in Fig 4E. The plasmid used should be mentioned somewhere.
- A description of the supplementary tables should be provided.
- Within the supplementary table containing the tabs "all proteins", "regulated", "unique IFNg", "Sheet4" and "Unique resting", the contains of the tabs don't match their title (ie, "unique IFNg" and "Unique resting" seem to have been exchanged).
- Other points: Quotes in the introduction do not always fit the previous sentence (ie, line 45 "brown et al", line 54 "Jain et al"). The author should double-check that appropriate reference are used throughout the text.

- Additional non-essential suggestions for improving the study (which will be at the author's/editor's discretion):

- Depending on how the authors develop the next version of this manuscript, they may consider moving Fig 3D-F after Fig 4-5, as Fig 4-5 focus on the impact of RNF115 loss but do not explore the role of its potential downstream target.
- The supplementary Fig 3 could be split into 2 supplementary figures, one including panels A-B and the other including panels C-D. The panels C-D could be moved later in the text.
- The authors have previously performed and published similar proteomic investigations, which are quoted in the text. They may clarify that all datasets displayed in this study have been freshly generated.

Referee #4 (Report for Author)

In this study, the authors investigate post-translation regulation of phagosomal maturation in macrophages by ubiquitylation of phagosomal-relevant vesicle trafficking proteins. Specifically, the authors demonstrate that i) phagosomal proteins in IFN-y-treated macrophages have increased ubiquitylation on specific lysine residues; ii) RNF115 is one such phagosomal-localized E3 ligase that negatively regulates phagosomal maturation through its ubiquitin ligase activity, and iii) regulation of phagosomal maturation by RNF115 has important repercussions in the inflammatory

response against pathogenic bacteria such as, S.aureus. Currently in the literature, it is unclear as to how IFNy treatment delays phagosome maturation in macrophages. However, the authors provide one explanation through ubiquitylation. Although ubiquitylation has been determined in previous studies to play a role in phagosome maturation, the authors have identified RNF115 as a specific E3 ligase that regulates this process and further characterized its role both in vitro and in vivo.

Overall, the data is novel, convincing, and high quality. The evidence provided adequately supports the authors major conclusions, with a few exceptions pertaining to the effect of ubiquitylation on phagosomal trafficking proteins and the direct ubiquitylation of phagosomal proteins by RNF115 (see major points). The greatest weakness of the study is that the authors demonstrate 'what' ubiquitylation does to phagosome maturation but very limitedly, 'how'. While the authors convincingly show RNF115's ubiquitin activity delays phagosome maturation in macrophages, it is unclear if this is due to the inability of relevant proteins to traffic to the phagosome (ie. protein recruitment is disrupted by ubiquitylation of proteins on the phagosomal membrane), phagosomes cannot acquire lysosomal proteins to mature (ie. ubiquitylation of phagosomal and lysosomal fusion proteins prevent phagosome-lysosome fusion due to disrupted protein interactions), both or through an alternative mechanism.

Major points:

1. Supplementary figure 2. This provides a rational argument as to how ubiquitylation negatively regulates phagosome maturation as phagosomes may not be able to fuse with lysosomes due to disrupted SNARE interactions. Firstly, I recommend moving this supplementary figure to the main paper. Secondly, it would strengthen the study to explore the effect of RNF115 KO on SNARE interactions through pulldown and/or colocalization etc. +/- INFg.
2. Can phagosomal fusion proteins localize to phagosomes due to ubiquitylation? In Figure 3F, I would like to see the proteomic data on lysosomal fusion proteins shown in Supplemental figure 2.
3. Although the authors show that ubiquitylation of phagosomal proteins in increased in IFNy-treated macrophages, RNF115 has not been demonstrated to directly ubiquitinate phagosomal proteins. To strengthen this claim, phagosomal ubiquitylation should be characterized in WT VS RNF115 KOs, such as Figure 1C, D, E, F and G.

Minor points:
1. Line 58. May be helpful to include a brief description of the roles of the E1, E2 and E3 ubiquitin enzymes.
2. Line 61. Missing K27. Clarify that methionine is the N-terminus. Better to rearrange the order of the residues and N terminus to match the text.
3. Figure 3F. I am not convinced that there is no significance between the WT and RNF115KO for proteins Ap3d1 and Chmpb1 yet almost every lysosomal protein has significance.
4. Figure 4. Why does 4B and 4C have shaded areas for SEM but not 4A? In 4B, it looks as though there are 4 lines mid-graph (ie. the lines representing WT+Bafi and RNF115 KO+Bafi). This should be fixed for clarity. Further, in 4A and 4C, it may be better to change "KO" in the graph legend to "RNF115 KO" like the rest of the graph legends in the figure for clarity.
5. Line 289. Clarify that phagosomes fluoresce when the lumen has high proteolytic activity, not due to acidity like pHrodo.
6. Lines 523 and 527. Was DQ red BSA used or green (see line 289)?
7. Lines 630 and 650: TSB abbreviation used twice.

Please find attached the initial rebuttal to the questions raised by the reviewers. We feel we can do many of the experiments, however, as always, they may not provide the answers the reviewers are looking for.

The main experiments that we will do are:

· A GlyGly-pulldown of ubiquitylated peptides from magnetic phagosomes from WT and RNF115 KO cell line macrophages followed by quantitative proteomics. This will hopefully give some evidence about RNF115 substrates and therefore a better insight into the mechanism. We can only really use magnetic phagosomes (which are less pure than latex phagosomes) as we will require hundreds of ug per replicate. With latex beads this would amount to ~2000 cell culture dishes which is rather unpractical. Any findings will focus on known phagosomal proteins. As you certainly know, proving that a protein is a direct substrate of an E3 ligase, is rather challenging.

· We will perform latex phagosome proteomics of WT, RNF115 KO and KO complemented with the "ligase dead" (actually E2-non-binding) mutant W259A. this will show that the ligase activity is required for the effect (although we are convinced that our complementation experiments showing these effects in the phagosome maturation assay already indicate this.

· We will perform time course experiment of phagosomes 30 min/0', 30'/30' and 30'/180' in resting and IFN-g activated macrophages and Western Blots to show retention of ubiquitin over the time course.

· We will do infections with S. aureus pH rodo, +/- gentamycin, +/- IFN in wt and RNF115 KO cells to show by another way that intraphagosomal bacteria are degraded faster in the KO. We will try similar experiments with Salmonella and the more cytosolic SifA mutant, but these are difficult experiments and may not work.

Otherwise, we will take up most of comments by the reviewers. I apologise for the many mistakes in the manuscript, it probably shows that we are all almost burnt out by the time we submitted the manuscript.

We will not perform additional in vivo experiments as we feel they likely won't add much to the manuscript considering the relatively modest effect seen in previous experiments and as these experiments would take another ~6 months.

Referee #1 (Report for Author)

The manuscript EMBOJ-2021-108970 by Bilkei-Gorzo et al. describes the proteomic analysis of phagosomes in resident and in IFNg stimulated macrophage-like cells. Whereas the authors' previous work was more concentrated on the general composition of phagosomes in resident and activated cells, they now focus on quality and quantity of protein ubiquitylation on phagosomes under either condition. Using sophisticated mass spectrometry and immunoblotting, they describe for the first time the diversity of ubiquitin chain links on phagosomes vs complete cell lysates and they identify five E3 ubiquitin ligases that are present at increased concentrations after macrophage activation. One of them, RNF115, was studied in more detail and the authors provide evidence that this ligase promotes phagolysosome formation in activated cells and that it participates in the down-regulation of pro-inflammatory cytokines during infection. Therefore, this study is the first to connect a specific E3 ligase to phagosome immune effector functions. Overall, this is a well-done

study which furthers the molecular understanding of phagosome maturation which will stimulate many scientists to consider postranslational protein modifications in general and ubiquitylation in particular as important regulators of phagosome development. A number of points need to be addressed:

Major comments

1. The Supplemental Tables are not self-explanatory. In fact they did not come with any text. Please include instructive table legends for the uninitiated readers in the supplemental information section.

Will be done.

2. The authors use vimentin as a loading control (e.g., Fig 1C-E). However, vimentin concentrations on phagosomes change with maturation as do, understandably, the concentrations of EEA1 (Fig. 1D). How can it then be used as loading control (l. 154)? Then, in Fig S1B, vimentin is used as a control protein indicating that luminal proteins are not digested during treatment with the deubiquitinase USP2. I understand that the fact that there are normal vimentin concentrations in the treated sample is interpreted as USP2 did not act as a general protease. But why then is vimentin, a cytosolic cytoskeletal protein, present in the lumen of phagosomes ("in" in Fig S1B)? And, thirdly, why then does "the presence of vimentin on the phagosomal membrane demonstrate(d) that phagosomes remained intact", i.e., show that they are not permeabilized (l. 139-140)?

Explain the difficulty of loading controls on phagosomes. Adding Ponceau stains as loading control.

3. With reference to the infection experiments shown in Fig. 4E: This is a flow-cytometry experiment but no "Materials and Methods" were given. One big disadvantage of a flow cytometry experiment for intracellular bacterial killing is that bacteria attached to macrophages will yield a signal even when killed by antibiotics. Second, it would have been helpful not to only see the 6 h incubation result but also the initial ("0 h") starting live bacterial cell number which would help to evaluate whether multiplication and/or killing occurred (differentially). Third, the datasets for the same experiment but from IFNg-activated BMA cells is needed as this manuscript centers around an E3 ligase that is more concentrated on phagosomes in activated vs resident macrophages. Forth, a much more established experiment to quantify live intracellular bacteria at times 0 and 6 h of infection is the 'gentamicin protection assay'. Actually, the authors have described this technique in their Materials-and-Methods section (l. 645-653) but there is no corresponding data set in the manuscript. This would be the perfect experiment to use using samples with and without IFNg activation at 0 and 6 h of infection. Fifth, the authors should make clearer that Listeria is not the paradigm cytosolic pathogen but that other intracellular pathogens (Shigella, Francisella, Rickettsia and more) also break their phagosome membranes at various times of infection which may expose them much longer than Listeria to the action of RNF115 before they actually break out. Vice versa, S. aureus is taken by the authors as a paradigm for phagosomal pathogens but like all intra-host cell phagosomes/vacuoles the S. aureus phagosome is unique in its composition and certainly not representative for all bacteria/pathogens. Conclusions are to be made cautiously and not generally ("vacuolar" vs "cytosolic" pathogen). It would have been more revealing if a non-replicating, intra-phagosomal bacterium would have been used instead of S. aureus, such as apathogenic Listeria innocua which shares many features with L. monocytogenes but lacks all 'real' virulence factors.

Replication with *S. aureus* pH rodo, +/- gentamycin, +/- IFN in wt and RNF115 KO cells.

4. Re. the experiment in which the authors show that ubiquitination can dramatically affect the recognition of a cellular protein by an antibody (a very interesting side aspect of the paper): L. 211 states "as this is the area against which antibodies were raised". It is likely "against which our antibodies were raised" making this a more special than a general observation? But, more importantly it should be mentioned that the antibodies used here were raised against a synthetic peptide which therefore could not be modified vs an authentic mammalian protein which could be ubiquitinylated. Still, how the conclusions were obtained, remains unclear because: (1) The ubiquitination sites on Rab7 in vivo are K38, K191 and K194 (Modica and Lefrancois, Small GTPases 11: 167) and (2) the monoclonal antibody from Cell Signaling Technologies which was used here is raised to a "peptide corresponding to residues surrounding Glu188 of human Rab7 protein." So one must assume that the residues K191 and K194 were relevant here and that the antigenic peptide only become visible after deubiquitination. But then there would be no higher-molecular weight ubiquitinated Rab7 forms to be detected by the antibody: Ubiquitin there: No antibody binding - ubiquitin gone: Antibody binding but no higher molecular weight forms. Please explain!

Will explain better.

5. The effect of RNF115 knockout on phagosome acidification (l. 297; Fig. 4B) is minor and its evaluation therefore depends even more than usually on the statistics approach used. Here, "error bars were generated from standard error of the mean of 6 replicates". Please indicate whether these were biological replicates (independent experiments) or whether these were technical replicates (parallel samples) which would not be adequate (Vaux et al., EMBO Reports, 2012). Also consider the fact that the zero time data of the RNF115 KO cells is already somewhat higher than for WT.
On a similar note, the experiment in Fig 1 F has been performed 5 times with 3 technical replicates per treatment. The authors state that due to the inherent variability of this complex experiment they combined all of the data acquired, yielding 15 (5 x 3 technical replicates) data points per condition. However, the way use these data, each technical replicate is considered an independent experiment. This is misleading, when it comes to determination of the standard error of the mean (SEM): SEM depends on sample size n (n, biological replicates) which here is actually 5, not 15. Since the authors show all of the data points, the variability becomes obvious even without SEM. Moreover, as t test p-values depend on SEM and sample size n, increasing the sample size to 15 may erroneously indicate that the differences observed are statistically significant. The authors should rather calculate means of the technical replicates for each of the independent experiments and show these instead (5 data points = means per condition), as in Fig 1 G.

Will change to 5 data points.

7. l. 97: "Total ubiquitylation stays almost constant over the whole phagosomal maturation process of both resting and IFNg-treated macrophages (Figure 1 D)." Fig 1 D only shows the kinetics of acquisition of ubiquitylated proteins by phagosomes in resting cells.

Will do the experiment. Resting + IFN WT BMDMs 30’/0, 30’/30’, 30’/180’

Minor comments
8. l. 83, "delays" instead of "reduces"

9. l. 91, "pure" instead of "highly pure", l. 144: "abundant" instead of "highly abundant", l. 200 "ubiquitylated" instead of "highly ubiquitylated", particularly as it is hard to see how the level of

ubiquitination was determined by the techniques used here.

10. l. 113-114, Figs 1F and 1G mixed up?

11. l. 261: CRISPR/Cas9 editing of RAW264.7 cells: Not mentioned in Materials and Methods, only for BMA cell line.

12. l. 264: "To determine whether RNF115 is involved in the regulation of phagocytosis": Was there reason to believe it would be?

13. l. 399: Please explain "ISGylation"

14. l. 410 and 1066: Is there precedence of SNARE protein motifs being inactivated by SNARE motif ubiquitylation or is this just speculation?

15. l. 499: The description of the recombinant proteins used in this study is extremely brief. Are all or any of these proteins mouse enzymes? It would be helpful to have database accession numbers for the sequence/identity or literature reference(s). Which was the E. coli strain used?

16. l. 576: Most other experiments were made using RAW264.7 cells. Please explain why an alternative cell line was used here.

17. Mat & Meth: What kind of cells are the RAW264.7 and BMA3.1A7 cell lines used here? Please explain in the main text.

18. Fig 4G, the ELISA experiment needs to be described in more detail, both in the figure legend and the material and methods section (experimental details, sample sizes, biological versus technical replicates).

19. l. 103, the ubiquitin-chain type specific antibodies are not specified in main text, figure legend, or Material and Methods section. Further information would be appreciated.

20. Some E3 ligases are enriched in phagosomes from IFNg-treated cells over phagosomes from untreated cells. However, in Fig 2 C it reads "IFNg only" for these E3 ligases, suggesting that these E3 ligases were absent from resting cell phagosomes. What is the threshold for the authors to define an E3 ligase as enriched?

21. The introduction should include a few sentences about IFNg and its relevant effects on macrophages and phagosome biogenesis.

22. Fig 1C/D: The authors should comment in the main text or figure legend how phagosome and TCL sample concentrations were adjusted to each other. This is especially important for the conclusion that ubiquitylated proteins are enriched on phagosomes as compared to the TCL.

23. Fig 1 F: 'M1' is not specified, in Figure 1 H 'UT' is not specified

24. Fig 1I: A scale bar is missing. Moreover, a brightfield image would be appreciated to show the position of the phagocytosed silica bead. A brightfield image should be included in Fig 3 C, too.

25. Fig 2F legend reads: "Level of shading of edges indicates interaction confidence." It rather seems as if the shading of the connecting lines indicates interaction confidence.

26. Axis titles should describe more directly what the authors have measured. This applies especially to Fig 5.

Additional non-essential suggestions for improving the study

27. The authors show here that they have the technology to identify ubiquitinylated proteins and that they have identified an E3 ligase which is required to exert immune functions during activation. It would be very interesting to see which phagosomal proteins precisely are ubiquitinylated by RNF115 in activated versus non-activated macrophages and in RNF115 WT versus KO cells, particularly as this may identify some obvious and interesting further candidates to follow up.

GlyGly pulldown of magnetic phagosomes followed by proteomics.

Referee #2 (Report for Author)

In the manuscript by Mathias Trost and colleagues, the authors investigated the role of RNF115, a ubiquitin ligase of the RING Finger family, in regulating phagosomal function. The authors show that RNF15 acts as a classical E3 ubiquitin ligase that it affects ubiquitylation of phagosome associated proteins and subsequently regulates innate responses to S.aureus in vitro and in vivo.

Strengths of the manuscript

The discovery of RNF115 to be an E3 ligase involved in modulating phagosome maturation is novel. In particular, it seems to delay dynamics of maturation. ubiquitylation of vesicle transport related proteins, some of which might be direct substrates.
RNF115 mediated ubiquitylation directly affects S.Aureus infection dynamics, demonstrating a role of ubiquitylation in the associated innate immune response.
These are all new findings are are worth publishing. In addition, the manuscript is well written, and the experiments have been carried out and are described with a high technical standard.

Weaknesses of the manuscript

Figure 2 shows the GlyGly modified proteins described in this study. The authors show that also components of the UBL system, in particular ISG15, appears to be enriched upon IFNgamma induction in their cellular system. As Ub and ISG15 share the same C-terminal sequence, ISG15ylated and Ubiquitylated proteins, upon trypsin digestion, result both in GlyGly-modified peptide fragments.

Therefore, there should be some caution in assigning all GlyGly-K sites identified to modifications by ubiquitin (e.g. Figure 2F). Granted, ISGylation is
predominantly induced by IFNalpha/beta, but this might be intertwined as the authors show with increased levels of Herc6 (Figure A), an E3 ligase that might conjugate ISG15 rather than ubiquitin (not completely established yet). Therefore, it is recommended that the authors should carefully compare their GlyGly proteomics data to know ISG15ylation signatures, now readily available in the literature.

Will compare datasets and describe the possibility of sites being ISGylation sites better.

K11 ubiquitylation seems the linkage type mostly accumulating upon IFNgamma exposure. This appears to be an interesting observation. The authors do not really follow up on their Ub-linkage characterisation (Figure 1) in the experiments shown in the rest of the manuscript. Also, the authors state the effect of RNF115 on the ubiquitylation status of a panel of phagosome associated proteins without going into direct enzyme-substrate relationships. However, the latter may require substantial additional work and maybe outside the scope of this work.

GlyGly pulldown of magnetic phagosomes followed by proteomics.

There appears to be a discrepancy between RNF115 delaying phagosome maturation, but at the same time, enhancing immune responses that involved phagosome mediated microbial uptake. Also, reduced inflammation/tissue damage upon RNF115 removal, as shown by the authors in Figure 5, does not seem to be consistent with enhanced capacity to clear microbial threats. Could the authors further elaborate on how RNF115 fine-tunes phagosome ubiquitylation to achieve this?

Discuss the complexity of *in vivo* data. Expression pattern of RNF115 in cells/tissues. More molecular understanding through Gly-GLy-pulldown.

In summary, this is a novel and interesting study that, upon addressing the concerns listed above in a convincing manner, should be considered for potential publication.

Referee #3 (Report for Author)

- General summary and opinion about the principal significance of the study, its questions and findings:

In this study, Bilkeu-Gorzo et al. display a sequence of 3 quantitative proteomic analyses to investigate the regulation of the phagosome ubiquitinome. First, they compare the ubiquitylation of isolated phagosomes with the total cell lysate (TCL) in the presence or absence of IFN-gamma and demonstrate increased ubiquitylation of phagosomes upon IFN-gamma activation. Besides, they identify the ubiquitin chains highly present in phagosomes compare to TCL and increased upon IFN-gamma activation. Second, they analyze the proteome and ubiquitinome of isolated-phagosomes. They showed an increased abundance of E1, E2, and E3 ligases at the phagosome upon IFN-gamma activation, as well as increased ubiquitylation of innate immunity and vesicle trafficking proteins. Focusing on one of the E3 ligases, RNF115, they perform a third proteomic analysis comparing the phagosome proteome of WT and RNF115 KO cells and show that loss of RNF115 affects the abundance of many proteins at the phagosome. They do not investigate further these proteins, nor confirmed those hits. Finally, they observe that loss of RNF115 affects phagosome proteolysis and acidification, as well as immune response during bacterial infection in macrophages and in vivo. The mechanism is not addressed.

The quantity of proteomic information gathered in this study is consequent and contains a true potential for a better understanding of phagosome biology, which is the strong point of the study.

Yet, while many proteins are identified, the data are not integrated to reach a mechanistic understanding of the role of (IFN-gamma-induced) ubiquitylation of the phagosome. Many additional experiments should be implemented to test the hypothesis opened by the proteomic approaches. In the manuscript's current state, the mechanistic conclusions are mostly overinterpreted /not supported by the data.

- Specific major concerns essential to be addressed to support the conclusions:

1_ Additional experiments should be performed to provide a mechanistic understanding of the role of IFN-gamma-induced ubiquitylation of the phagosome. Notably, the link between IFN-gamma activation and the function of RNF115 in phagosomal ubiquitylation and maturation should be established (for instance, +/- IFN-gamma conditions may be included from Fig 4b onward and the ubiquitin chain affected by RNF115 in the presence/absence of IFN-gamma may be tested). In particular, the results obtained with RNF115 KO should be tested with the RNF115 W259A mutant in order to conclude on the role of RNF115 ubiquitylation activity. To capitalize on the results obtained by the third proteomic analysis (RNF115 KO vs WT), the authors should confirm some of the most promising hits identified and test their recruitment to the phagosome under RNF115 W259A expression. Finally, the study would highly beneficiate from linking one of the proteins (or complexes) significantly depleted or enriched at the phagosome upon RNF115 KO with the RNF115-controlled phagosome maturation.

Will perform WT vs RNF115 KO and RNF115 W259A phagosome proteomics.

2_Regarding the infection experiments, the experimental setting does not support the conclusion that RNF115 KO increases degradation of phagosomal S. aureus but not cytosolic Listeria because the bacterial localizations are not tested. Indeed, the localization of S. aureus and L. monocytogenes are predicted from the literature but not shown by the data. It is now widely established that despite the main cytosolic lifestyle of L. monocytogenes, a subpopulation of infected cells contains vacuolar Listeria. Besides, as mentioned by the authors, S. aureus is mainly extracellular and only a small number of them are internalized into phagosomes. As the authors extrapolate on the link between RNF115 effect and the pathogen intracellular localization ("This suggests that loss of RNF115 affects specifically innate immune signalling from the phagosome, while signalling from the cytosol is unaffected." Lines 340-342), the experimental design should minimize all differences in the pathogenicity other than bacteria localization (avoiding divergent effects of species-specific bacterial effectors). An alternative would be to use bacteria that can display both cytosolic and vacuolar lifestyles such as Salmonella. Here a fluorescent reporter of the bacteria localization would be particularly suited for the authors' flow cytometry approaches. The cytosolic and vacuolar subpopulations can be easily enhanced using mutant strains.

We will try flow cytometry experiments with Salmonella strains which have specific reporters for acidification and other cellular responses. Use of SifA mutant (described by Holden lab) which enhances cytoplasmic localisation. Not sure if these experiments will work, but we will try.

3_ Generating RNF115 KO mice is a great asset for in vivo investigations of the role of RNF115 during bacterial infections. Yet, this tool is barely exploited in the manuscript and does not support the conclusion that RNF115 enhances bacterial clearance. In particular, the authors should quantify the number of bacteria and not restrict their analysis to the liver only. In addition, performing bacteria subcutaneous/local injection rather than intravenous injection may be more appropriate to quantify tissue damage.

We don't believe that further in vivo experiment will enhance the manuscript considering the modest effect seen in the infection model. We will add data from the spleens of the infected animals, currently only livers shown.

4_ The current version of the manuscript contains numerous mistakes in the text and figures. Individually taken, these concerns are minor, but due to their high number, they significantly impair the understanding of the manuscript and the trust in the data quality. The authors should make a thorough check of the figures, main text, and captions to correct and clarify any similar problem in addition to the one listed in the section below.

- Minor concerns that should be addressed (listed in order of appearance in the manuscript):

- Fig 1D: the text says "both resting and IFN-gamma treated macrophages" but only one condition is displayed and no information regarding the IFN-gamma treatment appears in the caption.
- Fig 1D: Why is the vimentin band unequal between conditions?
- Fig 1E: EEA1 should be included as a purity marker like in Fig 1C-D.
- The text should include a note on the fact that IFN-gamma stimulation does not significantly affect phagocytosis (ie, the number of phagosomes formed is similar with/without IFN-gamma).
- Fig 1H: "UT" should be explained in the caption or text.
- The introduction of the different DUBs specificity lines 116-119 and Fig 1H lacks clarity and is sometimes inconsistent between text and figure. The meaning of the signs ">" and ">>" should be clearly stated. A suggestion would be to replace the top left box of Fig 1H with a table crossing the mentioned DUBs and their targets.
- Fig 1.I : A panel showing the full cell (like in Fig 3C) should be included.
- Fig 1.I: The scale bar is missing.
- Fig. 1I: Why beads of 3 microns of diameters are used here while the size used for phagosome isolation is 0.8 micron?
- The caption of Fig 1C-E should be untangled. It is currently hard to follow.
- In the paragraphs contained between lines 168-190, most references to Fig 2 subpanels are incorrect.
- Fig 2F is not described in the text.
- Supplementary Fig 2B is not described in the text.
- In Supplementary Fig 2, what do the blue and red parts of the SNARE sequences correspond to? This should appear in the caption. Besides TAB2 PD should be introduced in the caption (ie, "TAB2 PD is a K63-chain binding protein").
- Lines 220, 223, and Fig 3E, the term "upregulated" is incorrect and should be replaced by "enriched" as it refers to protein abundance on a subcellular compartment.
- Fig 3A: The results displayed are confusing in regard to the previous figures. My understanding is that the E3 ligases plotted in Fig 3A are selected from the list plotted in Fig 2C (this information, if correct, should appear somewhere in the text or caption, as well as the selection criteria). However, why Herc6 and RNF115 are written "IFN only" in Fig 2C - suggesting that they were below the detection level without IFN stimulation - but their log2 intensity in the unstimulated condition is display in Fig 3A?
- Fig 3A: The carried t-tests show 4 stars in the comparison of RNF115 enrichment between Ctrl and IFN-gamma treatment but only 2 stars for Herc6 (which by eye look similar). To dissipate any misunderstanding, the authors may provide the raw data used for this figure in their rebuttal.
- Fig 3B: Rab7 cannot be used as a purity control as it interacts with RNF115 (as explained in lines 228-231) and should be removed from the plot.
- Fig 3C: a comparison with RNF115 recruitment at the phagosome without IFN activation would be relevant, and quantification would make the results more convincing.

- Fig 3D: The proteins named within the volcano plot are not mentioned in the text or caption. It would be preferable to point at proteins that are later mentioned in the text (such as the one present in Fig 3F).
- The results displayed in Supplementary Fig 3C-D are described lines 263-268 as such: "Ablation of RNF115 or loss of E2 binding of RNF115 did not affect phagocytic uptake of carboxylated particles (supplementary figure 3C) or bacterial uptake (supplementary figure 3D)." Yet, RNF115 mutant is only introduced in line 301, and the bacteria in line 327. I believe that the authors may have wrongly reshuffled the position of this line in their manuscript. This needs to be re-organized (see also additional suggestions).
- In Supplementary Fig 3: Does "RFU" stands for "relative fluorescence units" and "CytD" for "CytochalasinD"? This should appear in the caption. Similarly, the reason why CytochalasinD is used (I guess as a negative control ?) should be stated somewhere. The material and method section describes CFU determination but this does not appear in any figure. Maybe the authors meant "CFU" instead of "RFU" in the supplementary Fig 3D?
- Supplementary Fig 3D: The condition "RNF115 W259A" should be included.
- Lines 278-279: the statement "loss of RNF115 also decreased recruitment of the ESCRT complex highlighting the role in ubiquitylation for the functional role of the complex" is not supported by the data because RNF115 is depleted but not replace by RNF115 W259A. Additional experiments should be performed or the statement should be corrected. Same comment for line 290 " this data shows that ubiquitylation plays an important role in protein and vesicle trafficking."
- Line 283, the authors wrote "TRAPP was significantly less abundant on RNF115 KO phagosomes and may explain the retention of cargo that was meant to be secreted". Was this retention of cargo observed? If yes, the authors should include such data or references to published work.
- Fig 4A: the result of IFN-gamma treatment is not commented in the text.
- Fig 5B: what is Bafilomycin used for? This and the interpretation of the results should appear in the text (or be removed from the figure).
- Fig 5B: The conditions +/-IFN gamma treatment should be included.
- Fig 5B: Strange lines appear around "WT+Bafi" and "RNF115+Bafi" lines instead of shaded area representing SEM.
- Lines 298-300: " These data were further validated by complementing RNF115 KO with RNF115-HA that reversed enhanced phagosomal proteolytic activity of KO macrophages". Where are the data?
- Lines 335-336: "The analysis revealed that RNF115 KO deficiency did not impact uptake of either pathogen" refers to results displayed in Supplementary Fig 4D, not Fig 4E. The following sentence "However, degradation of phagosomal S. aureus was significantly increased in the KO, while cytosolic Listeria were not affected" most likely refers to Fig 4E.
- Fig 4E: the "non-infected" condition must be shown to determine the gate of GFP-positive signal versus autofluorescence.
- Fig 4E: the data doesn't allow to decipher if the lack of intracellular S. aureus is due to bacterial clearance or lack of replication. Several time points should be displayed to provide this information.
- Lines 366-367: the link between CD68-stained macrophage and tissue repair should be justified by literature references.
- Line 368: the conclusion on bacterial clearance is not supported by the data as the bacteria have not been quantified.
- Fig 5: Adding a condition with/without IFN-gamma stimulation would strengthen the results.
- In the material and methods, the bacteria are not described as fluorescent, but S. aureus GFP and L. monocytogenes eGFP are used in Fig 4E. The plasmid used should be mentioned somewhere.
- A description of the supplementary tables should be provided.
- Within the supplementary table containing the tabs "all proteins", "regulated", "unique IFNg", "Sheet4" and "Unique resting", the contains of the tabs don't match their title (ie, "unique IFNg" and "Unique resting" seem to have been exchanged).
- Other points: Quotes in the introduction do not always fit the previous sentence (ie, line 45 "brown

et al", line 54 "Jain et al"). The author should double-check that appropriate reference are used throughout the text.

- Additional non-essential suggestions for improving the study (which will be at the author's/editor's discretion):

- Depending on how the authors develop the next version of this manuscript, they may consider moving Fig 3D-F after Fig 4-5, as Fig 4-5 focus on the impact of RNF115 loss but do not explore the role of its potential downstream target.
- The supplementary Fig 3 could be split into 2 supplementary figures, one including panels A-B and the other including panels C-D. The panels C-D could be moved later in the text.
- The authors have previously performed and published similar proteomic investigations, which are quoted in the text. They may clarify that all datasets displayed in this study have been freshly generated.

Referee #4 (Report for Author)

In this study, the authors investigate post-translation regulation of phagosomal maturation in macrophages by ubiquitylation of phagosomal-relevant vesicle trafficking proteins. Specifically, the authors demonstrate that i) phagosomal proteins in IFN-y-treated macrophages have increased ubiquitylation on specific lysine residues; ii) RNF115 is one such phagosomal-localized E3 ligase that negatively regulates phagosomal maturation through its ubiquitin ligase activity, and iii) regulation of phagosomal maturation by RNF115 has important repercussions in the inflammatory response against pathogenic bacteria such as, S.aureus. Currently in the literature, it is unclear as to how IFNy treatment delays phagosome maturation in macrophages. However, the authors provide one explanation through ubiquitylation. Although ubiquitylation has been determined in previous studies to play a role in phagosome maturation, the authors have identified RNF115 as a specific E3 ligase that regulates this process and further characterized its role both in vitro and in vivo.

Overall, the data is novel, convincing, and high quality. The evidence provided adequately supports the authors major conclusions, with a few exceptions pertaining to the effect of ubiquitylation on phagosomal trafficking proteins and the direct ubiquitylation of phagosomal proteins by RNF115 (see major points). The greatest weakness of the study is that the authors demonstrate 'what' ubiquitylation does to phagosome maturation but very limitedly, 'how'. While the authors convincingly show RNF115's ubiquitin activity delays phagosome maturation in macrophages, it is unclear if this is due to the inability of relevant proteins to traffic to the phagosome (ie. protein recruitment is disrupted by ubiquitylation of proteins on the phagosomal membrane), phagosomes cannot acquire lysosomal proteins to mature (ie. ubiquitylation of phagosomal and lysosomal fusion proteins prevent phagosome-lysosome fusion due to disrupted protein interactions), both or through an alternative mechanism.

Major points:

1. Supplementary figure 2. This provides a rational argument as to how ubiquitylation negatively regulates phagosome maturation as phagosomes may not be able to fuse with lysosomes due to disrupted SNARE interactions. Firstly, I recommend moving this supplementary figure to the main paper. Secondly, it would strengthen the study to explore the effect of RNF115 KO on SNARE interactions through pulldown and/or colocalization etc. +/- INFg.

We are looking into possibly pulling down specific SNARE proteins from the RNF115 KO and WT and responses to IFN-gamma. However, these are difficult experiments and will need to do from total cell lysates.

Moving figure and maybe adding more insights.

2. Can phagosomal fusion proteins localize to phagosomes due to ubiquitylation? In Figure 3F, I would like to see the proteomic data on lysosomal fusion proteins shown in Supplemental figure 2.
Will highlight the data and move as suggested.

3. Although the authors show that ubiquitylation of phagosomal proteins in increased in IFNy-treated macrophages, RNF115 has not been demonstrated to directly ubiquitinate phagosomal proteins. To strengthen this claim, phagosomal ubiquitylation should be characterized in WT VS RNF115 KOs, such as Figure 1C, D, E, F and G.

GlyGly pulldown of magnetic phagosomes followed by proteomics.

Minor points:
1. Line 58. May be helpful to include a brief description of the roles of the E1, E2 and E3 ubiquitin enzymes.
2. Line 61. Missing K27. Clarify that methionine is the N-terminus. Better to rearrange the order of the residues and N terminus to match the text.
3. Figure 3F. I am not convinced that there is no significance between the WT and RNF115KO for proteins Ap3d1 and Chmpb1 yet almost every lysosomal protein has significance.
4. Figure 4. Why does 4B and 4C have shaded areas for SEM but not 4A? In 4B, it looks as though there are 4 lines mid-graph (ie. the lines representing WT+Bafi and RNF115 KO+Bafi). This should be fixed for clarity. Further, in 4A and 4C, it may be better to change "KO" in the graph legend to "RNF115 KO" like the rest of the graph legends in the figure for clarity.
5. Line 289. Clarify that phagosomes fluoresce when the lumen has high proteolytic activity, not due to acidity like pHrodo.
6. Lines 523 and 527. Was DQ red BSA used or green (see line 289)?
7. Lines 630 and 650: TSB abbreviation used twice.

1st Editorial Decision 17th Sep 2021

Thank you for tentative response to the referee reports, and proposal for revising this work for The EMBO Journal. I have now had a chance to consider these plans, and appreciate that especially the addition of the proposed Gly-Gly-pulldown/mass spec from magnetic phagosomes of RNF115 + vs - cells (and studying ligase-deficiency effects on latex-purified phagosome) should be helpful to add the deeper molecular understanding that had been missed by several referees in the original submission. Furthermore, the planned time course and additional cellular infection experiments would likely also address important reviewer concerns; while I agree that additional (new) in vivo work would not be the most salient point to incorporate.

Rebuttal of "The E3 ubiquitin ligase RNF115 regulates phagosome maturation and host response to bacterial infection", EMBO Journal EMBOJ-2021-108970R

We would like to thank the reviewers for their constructive criticism. We have added a lot of new data and hoped to respond to every point made by the reviewers.

Thank you for improving our paper!

Referee #1 (Report for Author)

The manuscript EMBOJ-2021-108970 by Bilkei-Gorzo et al. describes the proteomic analysis of phagosomes in resident and in IFNg stimulated macrophage-like cells. Whereas the authors' previous work was more concentrated on the general composition of phagosomes in resident and activated cells, they now focus on quality and quantity of protein ubiquitylation on phagosomes under either condition. Using sophisticated mass spectrometry and immunoblotting, they describe for the first time the diversity of ubiquitin chain links on phagosomes vs complete cell lysates and they identify five E3 ubiquitin ligases that are present at increased concentrations after macrophage activation. One of them, RNF115, was studied in more detail and the authors provide evidence that this ligase promotes phagolysosome formation in activated cells and that it participates in the down-regulation of pro-inflammatory cytokines during infection. Therefore, this study is the first to connect a specific E3 ligase to phagosome immune effector functions. Overall, this is a well-done study which furthers the molecular understanding of phagosome maturation which will stimulate many scientists to consider postranslational protein modifications in general and ubiquitylation in particular as important regulators of phagosome development. A number of points need to be addressed:

Major comments

1. The Supplemental Tables are not self-explanatory. In fact they did not come with any text. Please include instructive table legends for the uninitiated readers in the supplemental information section.

We have included that.

2. The authors use vimentin as a loading control (e.g., Fig 1C-E). However, vimentin concentrations on phagosomes change with maturation as do, understandably, the concentrations of EEA1 (Fig. 1D). How can it then be used as loading control (l. 154)? Then, in Fig S1B, vimentin is used as a control protein indicating that luminal proteins are not digested during treatment with the deubiquitinase USP2. I understand that the fact that there are normal vimentin concentrations in the treated sample is interpreted as USP2 did not act as a general protease. But why then is vimentin, a cytosolic cytoskeletal protein, present in the lumen of phagosomes ("in" in Fig S1B)? And, thirdly, why then does "the presence of vimentin on the phagosomal membrane demonstrate(d) that phagosomes remained intact", i.e., show that they are not permeabilized (l. 139-140)?

Due to the dynamic nature of the phagosome, a loading control is actually very difficult for phagosomes. We have tested over the years many different proteins and vimentin was one of the proteins that appear to not change significantly during maturation. We are not aware of the role of vimentin on phagosomes, however, it has been identified many times over the last 20 years of phagosomal proteomics and is actually rather abundant around the phagosome.
We think the reviewer may have misunderstood the experiment in Fig S1b. "ex" stands for the "supernatant" that is released from USP2 cleavage, while "in" stands for the intact phagosomes. As USP2 does not affect the association of vimentin to the phagosome, vimentin stays on the

phagosomes after USP2 treatment. We have changed the “ex” to “sup” to make this clearer and also changed the figure legend.

3. With reference to the infection experiments shown in Fig. 4E: This is a flow-cytometry experiment but no "Materials and Methods" were given. One big disadvantage of a flow cytometry experiment for intracellular bacterial killing is that bacteria attached to macrophages will yield a signal even when killed by antibiotics. Second, it would have been helpful not to only see the 6 h incubation result but also the initial ("0 h") starting live bacterial cell number which would help to evaluate whether multiplication and/or killing occurred (differentially). Third, the datasets for the same experiment but from IFNg-activated BMA cells is needed as this manuscript centers around an E3 ligase that is more concentrated on phagosomes in activated vs resident macrophages. Forth, a much more established experiment to quantify live intracellular bacteria at times 0 and 6 h of infection is the 'gentamicin protection assay'. Actually, the authors have described this technique in their Materials-and-Methods section (l. 645-653) but there is no corresponding data set in the manuscript. This would be the perfect experiment to use using samples with and without IFNg activation at 0 and 6 h of infection. Fifth, the authors should make clearer that Listeria is not the paradigm cytosolic pathogen but that other intracellular pathogens (Shigella, Francisella, Rickettsia and more) also break their phagosome membranes at various times of infection which may expose them much longer than Listeria to the action of RNF115 before they actually break out. Vice versa, S. aureus is taken by the authors as a paradigm for phagosomal pathogens but like all intra-host cell phagosomes/vacuoles the S. aureus phagosome is unique in its composition and certainly not representative for all bacteria/pathogens. Conclusions are to be made cautiously and not generally ("vacuolar" vs "cytosolic" pathogen). It would have been more revealing if a non-replicating, intra-phagosomal bacterium would have been used instead of S. aureus, such as apathogenic Listeria innocua which shares many features with L. monocytogenes but lacks all 'real' virulence factors.

We have removed that data and we have performed additional experiments with infections using Salmonella Typhimurium. *There is indeed an effect by any of the species that actively affect the phagosomal proteome. In order to make it more readable we have focussed the infection data now on Salmonella and S. aureus.*

4. Re. the experiment in which the authors show that ubiquitination can dramatically affect the recognition of a cellular protein by an antibody (a very interesting side aspect of the paper): L. 211 states "as this is the area against which antibodies were raised". It is likely "against which our antibodies were raised" making this a more special than a general observation? But, more importantly it should be mentioned that the antibodies used here were raised against a synthetic peptide which therefore could not be modified vs an authentic mammalian protein which could be ubiquitinylated. Still, how the conclusions were obtained, remains unclear because: (1) The ubiquitination sites on Rab7 in vivo are K38, K191 and K194 (Modica and Lefrancois, Small GTPases 11: 167) and (2) the monoclonal antibody from Cell Signaling Technologies which was used here is raised to a "peptide corresponding to residues surrounding Glu188 of human Rab7 protein." So one must assume that the residues K191 and K194 were relevant here and that the antigenic peptide only become visible after deubiquitination. But then there would be no higher-molecular weight ubiquitinated Rab7 forms to be detected by the antibody: Ubiquitin there: No antibody binding - ubiquitin gone: Antibody binding but no higher molecular weight forms. Please explain!

We feel this is indeed a very interesting part of the paper. Antibodies against Rab proteins are all raised against the C-terminal part of the proteins as the other areas of the proteins are too conserved around the GTPase domain.
We detected ubiquitylation sites for Rab7a on K126, K175, K191, K194. The reviewer is right that there should not be higher molecular forms. We believe that the big "blob" around 50k could be AMSH-LP itself as very abundant proteins sometimes give unspecific bands. We have highlighted that in the figure. The reviewer should also keep in mind that AMSH-LP is specific for K63 chains and therefore other chain types, perhaps not near the area against which the antibody was raised (K126/K171) may be other chain types than K63 and therefore be resistant against AMSH-LP treatment. This is supported by the data that there are individual bands looking like Rab7a + 1/2/3 ubiquitin molecules.
In the future, we will definitely make use of the Ubi-Crest approach to identify the different chain linkages on key proteins.

5. The effect of RNF115 knockout on phagosome acidification (l. 297; Fig. 4B) is minor and its evaluation therefore depends even more than usually on the statistics approach used. Here, "error bars were generated from standard error of the mean of 6 replicates". Please indicate whether these were biological replicates (independent experiments) or whether these were technical replicates (parallel samples) which would not be adequate (Vaux et al., EMBO Reports, 2012). Also consider the fact that the zero time data of the RNF115 KO cells is already somewhat higher than for WT.
On a similar note, the experiment in Fig 1 F has been performed 5 times with 3 technical replicates per treatment. The authors state that due to the inherent variability of this complex experiment they combined all of the data acquired, yielding 15 (5 x 3 technical replicates) data points per condition. However, the way use these data, each technical replicate is considered an independent experiment. This is misleading, when it comes to determination of the standard error of the mean (SEM): SEM depends on sample size n (n, biological replicates) which here is actually 5, not 15. Since the authors show all of the data points, the variability becomes obvious even without SEM. Moreover, as t test p-values depend on SEM and sample size n, increasing the sample size to 15 may erroneously indicate that the differences observed are statistically significant. The authors should rather calculate means of the technical replicates for each of the independent experiments and show these instead (5 data points = means per condition), as in Fig 1 G.

The PRM experiment was performed as such: phagosomes were independently isolated from 3 biological replicates (different passages of the cell line) and analysed by PRM. This has been done five separate times. They were not technical replicates such as injection of the same sample multiple times. We now use standard deviation as error bars.
As for the acidification assay: the assay has been performed with 6 samples in parallel and is representative of 4 experiments done independently. Unfortunately, the assay is usually providing more qualitative data and combining data obtained on different days is difficult. We made this clearer in the figure legend.
Please note that the data is supported by using the Salmonella Pasr reporter which responds to pH changes Fig 6D.

7. l. 97: "Total ubiquitylation stays almost constant over the whole phagosomal maturation process of both resting and IFNg-treated macrophages (Figure 1 D)." Fig 1 D only shows the kinetics of acquisition of ubiquitylated proteins by phagosomes in resting cells.

We have added a Western Blot showing the same data in IFN-g treated cells in Figure 1D.

Minor comments
8. l. 83, "delays" instead of "reduces"

Done.

9. l. 91, "pure" instead of "highly pure", l. 144: "abundant" instead of "highly abundant", l. 200 "ubiquitylated" instead of "highly ubiquitylated", particularly as it is hard to see how the level of ubiquitination was determined by the techniques used here.

Done.

10. l. 113-114, Figs 1F and 1G mixed up?

No, but reworded the sentence as it was confusing.

11. l. 261: CRISPR/Cas9 editing of RAW264.7 cells: Not mentioned in Materials and Methods, only for BMA cell line.

Now added.

12. l. 264: "To determine whether RNF115 is involved in the regulation of phagocytosis": Was there reason to believe it would be?

We test any phagosomal protein we work with if ablation leads to actual differences in phagocytosis as sometimes reported changes in maturation are mostly due to differences in phagocytosis.

13. l. 399: Please explain "ISGylation"
Added:
It needs to be noted that IFN-γ activation also increases phagosomal ISGylation, i.e. covalent addition of the interferon-induced ubiquitin-like modifier ISG15…

14. l. 410 and 1066: Is there precedence of SNARE protein motifs being inactivated by SNARE motif ubiquitylation or is this just speculation?

This is speculation. We added "likely"

15. l. 499: The description of the recombinant proteins used in this study is extremely brief. Are all or any of these proteins mouse enzymes? It would be helpful to have database accession numbers for the sequence/identity or literature reference(s). Which was the E. coli strain used?

We added this information. All the recombinant proteins are based on human enzymes and generated through MRC Reagents.

16. l. 576: Most other experiments were made using RAW264.7 cells. Please explain why an alternative cell line was used here.

During COVID19, when the laboratory was closed for several months, we lost many cell lines when our liquid nitrogen-cooled vessel was not maintained by university staff. We lost all RNF115 knock-out clones in RAW264.7 and had to re-do them. In the meantime, the lab had switched all their work

to the BMA cell line as it represents BMDMs better than RAW264.7 cells. So, we re-did the cells in BMA cells. All data shown in the paper is from these BMA cells and since most of the work is validated in BMDMs, we do not feel that this is a significant problem.

17. Mat & Meth: What kind of cells are the RAW264.7 and BMA3.1A7 cell lines used here? Please explain in the main text.

We highlighted in the methods that both cell lines are murine macrophage cell lines.

18. Fig 4G, the ELISA experiment needs to be described in more detail, both in the figure legend and the material and methods section (experimental details, sample sizes, biological versus technical replicates).

Added.

19. l. 103, the ubiquitin-chain type specific antibodies are not specified in main text, figure legend, or Material and Methods section. Further information would be appreciated.

Added.

20. Some E3 ligases are enriched in phagosomes from IFNg-treated cells over phagosomes from untreated cells. However, in Fig 2 C it reads "IFNg only" for these E3 ligases, suggesting that these E3 ligases were absent from resting cell phagosomes. What is the threshold for the authors to define an E3 ligase as enriched?

As this was a label-free proteomics experiment, “IFN only” or “Resting only” mean that these E3 ligases were only detected in the respective samples and not the other. Therefore, a ratio is difficult to generate unless imputation of the detection level is used. Added following sentence into the figure legend: “IFN only” or ”Rest only” are used when the proteins were only detected in the respective sample by label-free proteomics.

21. The introduction should include a few sentences about IFNg and its relevant effects on macrophages and phagosome biogenesis.

Added this.

22. Fig 1C/D: The authors should comment in the main text or figure legend how phagosome and TCL sample concentrations were adjusted to each other. This is especially important for the conclusion that ubiquitylated proteins are enriched on phagosomes as compared to the TCL.

Same protein amounts were used as determined by protein assays. We added this to the figure legend.

23. Fig 1 F: 'M1' is not specified, in Figure 1 H 'UT' is not specified

Added requested information in Figure legend 1.

24. Fig 1I: A scale bar is missing. Moreover, a brightfield image would be appreciated to show the position of the phagocytosed silica bead. A brightfield image should be included in Fig 3 C, too.

We added a new figure in figure 1L.

25. Fig 2F legend reads: "Level of shading of edges indicates interaction confidence." It rather seems as if the shading of the connecting lines indicates interaction confidence.

Changed it to "Level of shading of connecting lines indicates interaction confidence."

26. Axis titles should describe more directly what the authors have measured. This applies especially to Fig 5.

Done.

Additional non-essential suggestions for improving the study

27. The authors show here that they have the technology to identify ubiquitinylated proteins and that they have identified an E3 ligase which is required to exert immune functions during activation. It would be very interesting to see which phagosomal proteins precisely are ubiquitinylated by RNF115 in activated versus non-activated macrophages and in RNF115 WT versus KO cells, particularly as this may identify some obvious and interesting further candidates to follow up.

We have added this experiment in Figure 5.

Referee #2 (Report for Author)

In the manuscript by Mathias Trost and colleagues, the authors investigated the role of RNF115, a ubiquitin ligase of the RING Finger family, in regulating phagosomal function. The authors show that RNF15 acts as a classical E3 ubiquitin ligase that it affects ubiquitylation of phagosome associated proteins and subsequently regulates innate responses to S.aureus in vitro and in vivo.

Strengths of the manuscript

The discovery of RNF115 to be an E3 ligase involved in modulating phagosome maturation is novel. In particular, it seems to delay dynamics of maturation. ubiquitylation of vesicle transport related proteins, some of which might be direct substrates.
RNF115 mediated ubiquitylation directly affects S.Aureus infection dynamics, demonstrating a role of ubiquitylation in the associated innate immune response.
These are all new findings are are worth publishing. In addition, the manuscript is well written, and the experiments have been carried out and are described with a high technical standard.

Weaknesses of the manuscript

Figure 2 shows the GlyGly modified proteins described in this study. The authors show that also components of the UBL system, in particular ISG15, appears to be enriched upon IFNgamma induction in their cellular system. As Ub and ISG15 share the same C-terminal sequence, ISG15ylated and Ubiquitylated proteins, upon trypsin digestion, result both in GlyGly-modified peptide fragments.

Therefore, there should be some caution in assigning all GlyGly-K sites identified to modifications by ubiquitin (e.g. Figure 2F). Granted, ISGylation is predominantly induced by IFNalpha/beta, but this might be intertwined as the authors show with increased levels of Herc6 (Figure A), an E3 ligase that might conjugate ISG15 rather than ubiquitin (not completely established yet). Therefore, it is recommended that the authors should carefully compare their GlyGly proteomics data to know ISG15ylation signatures, now readily available in the literature.

This is a good point and we do discuss it in the discussion. Since ubiquitin is so much more abundant (~60-fold) than ISG15, we believe that the majority of sites will be ubiquitylation sites and not ISG15. We have added to the discussion to highlight the fact that some sites could be ISGylation.

K11 ubiquitylation seems the linkage type mostly accumulating upon IFNgamma exposure. This appears to be an interesting observation. The authors do not really follow up on their Ub-linkage characterisation (Figure 1) in the experiments shown in the rest of the manuscript. Also, the authors state the effect of RNF115 on the ubiquitylation status of a panel of phagosome associated proteins without going into direct enzyme-substrate relationships. However, the latter may require substantial additional work and maybe outside the scope of this work.

We agree that this is very interesting indeed. This paper is an initial study on the role of ubiquitylation on the phagosome and we plan to do more experiments characterising the role of individual chain linkages on the phagosome.
We have added data on identifying potential substrates for RNF115 in Figure 5.

There appears to be a discrepancy between RNF115 delaying phagosome maturation, but at the same time, enhancing immune responses that involved phagosome mediated microbial uptake. Also, reduced inflammation/tissue damage upon RNF115 removal, as shown by the authors in Figure 5, does not seem to be consistent with enhanced capacity to clear microbial threats. Could the authors further elaborate on how RNF115 fine-tunes phagosome ubiquitylation to achieve this?

This is a difficult question. Generally, we believe that changes to the phagosome maturation will also affect the time receptors such as TLRs will signal from the phagosome. However, it will also affect the bacterial response to the increased acidification as our data shows for Salmonella. We believe some of the data in Figure 5 gives some details how RNF115 is mechanistically changing the phagosome function.

In summary, this is a novel and interesting study that, upon addressing the concerns listed above in a convincing manner, should be considered for potential publication.

Referee #3 (Report for Author)

- General summary and opinion about the principal significance of the study, its questions and findings:

In this study, Bilkeu-Gorzo et al. display a sequence of 3 quantitative proteomic analyses to investigate the regulation of the phagosome ubiquitinome. First, they compare the ubiquitylation of

isolated phagosomes with the total cell lysate (TCL) in the presence or absence of IFN-gamma and demonstrate increased ubiquitylation of phagosomes upon IFN-gamma activation. Besides, they identify the ubiquitin chains highly present in phagosomes compare to TCL and increased upon IFN-gamma activation. Second, they analyze the proteome and ubiquitinome of isolated-phagosomes. They showed an increased abundance of E1, E2, and E3 ligases at the phagosome upon IFN-gamma activation, as well as increased ubiquitylation of innate immunity and vesicle trafficking proteins. Focusing on one of the E3 ligases, RNF115, they perform a third proteomic analysis comparing the phagosome proteome of WT and RNF115 KO cells and show that loss of RNF115 affects the abundance of many proteins at the phagosome. They do not investigate further these proteins, nor confirmed those hits. Finally, they observe that loss of RNF115 affects phagosome proteolysis and acidification, as well as immune response during bacterial infection in macrophages and in vivo. The mechanism is not addressed.

The quantity of proteomic information gathered in this study is consequent and contains a true potential for a better understanding of phagosome biology, which is the strong point of the study. Yet, while many proteins are identified, the data are not integrated to reach a mechanistic understanding of the role of (IFN-gamma-induced) ubiquitylation of the phagosome. Many additional experiments should be implemented to test the hypothesis opened by the proteomic approaches. In the manuscript's current state, the mechanistic conclusions are mostly overinterpreted /not supported by the data.

- Specific major concerns essential to be addressed to support the conclusions:

1_ Additional experiments should be performed to provide a mechanistic understanding of the role of IFN-gamma-induced ubiquitylation of the phagosome. Notably, the link between IFN-gamma activation and the function of RNF115 in phagosomal ubiquitylation and maturation should be established (for instance, +/- IFN-gamma conditions may be included from Fig 4b onward and the ubiquitin chain affected by RNF115 in the presence/absence of IFN-gamma may be tested). In particular, the results obtained with RNF115 KO should be tested with the RNF115 W259A mutant in order to conclude on the role of RNF115 ubiquitylation activity. To capitalize on the results obtained by the third proteomic analysis (RNF115 KO vs WT), the authors should confirm some of the most promising hits identified and test their recruitment to the phagosome under RNF115 W259A expression. Finally, the study would highly beneficiate from linking one of the proteins (or complexes) significantly depleted or enriched at the phagosome upon RNF115 KO with the RNF115-controlled phagosome maturation.

We have now added more experiments as requested, including usage of the W259A mutant and a digly-pulldown from RNf115 KO cells (Figure 5) where we identified potential substrates of RNF115 KO. While proving that they are true targets of RNf115 is difficult, the data provides some evidence about the mechanism of RNf115 function.

2_Regarding the infection experiments, the experimental setting does not support the conclusion that RNF115 KO increases degradation of phagosomal S. aureus but not cytosolic Listeria because the bacterial localizations are not tested. Indeed, the localization of S. aureus and L. monocytogenes are predicted from the literature but not shown by the data. It is now widely established that despite the main cytosolic lifestyle of L. monocytogenes, a subpopulation of infected cells contains vacuolar Listeria. Besides, as mentioned by the authors, S. aureus is mainly extracellular and only a small number of them are internalized into phagosomes. As the authors extrapolate on the link between RNF115 effect and the pathogen intracellular localization ("This suggests that loss of RNF115 affects specifically innate immune signalling from the phagosome, while signalling from the

cytosol is unaffected." Lines 340-342), the experimental design should minimize all differences in the pathogenicity other than bacteria localization (avoiding divergent effects of species-specific bacterial effectors). An alternative would be to use bacteria that can display both cytosolic and vacuolar lifestyles such as Salmonella. Here a fluorescent reporter of the bacteria localization would be particularly suited for the authors' flow cytometry approaches. The cytosolic and vacuolar subpopulations can be easily enhanced using mutant strains.

We have decided to remove the Listeria data entirely as it may confuse readers. We have added substantial amount of Salmonella data which highlights that the increased maturation affects Salmonella survival while also activating Salmonella's adaption response. In the short time within which we did these experiments Salmonella will be phagosomal. We believe other labs, with much better Salmonella expertise will be better suited to characterise the differences between cytoplasmic and phagosomal populations.

3_ Generating RNF115 KO mice is a great asset for in vivo investigations of the role of RNF115 during bacterial infections. Yet, this tool is barely exploited in the manuscript and does not support the conclusion that RNF115 enhances bacterial clearance. In particular, the authors should quantify the number of bacteria and not restrict their analysis to the liver only. In addition, performing bacteria subcutaneous/local injection rather than intravenous injection may be more appropriate to quantify tissue damage.

We have added more details about the phenotyping of the mice which was performed at the MRC Harwell lab. Unfortunately, our animal license does not allow us to do the suggest experiments.

4_ The current version of the manuscript contains numerous mistakes in the text and figures. Individually taken, these concerns are minor, but due to their high number, they significantly impair the understanding of the manuscript and the trust in the data quality. The authors should make a thorough check of the figures, main text, and captions to correct and clarify any similar problem in addition to the one listed in the section below.

We apologise for this and we have gone through the manuscript with great care.

- Minor concerns that should be addressed (listed in order of appearance in the manuscript):

- Fig 1D: the text says "both resting and IFN-gamma treated macrophages" but only one condition is displayed and no information regarding the IFN-gamma treatment appears in the caption.

We have now added a new western blot in Figure 1D with both IFNg and resting.

- Fig 1D: Why is the vimentin band unequal between conditions?

We have added LAMP1 as a marker showing the increase in maturation.

- Fig 1E: EEA1 should be included as a purity marker like in Fig 1C-D.

We have done that for figure 1C and LAMP1 for figure 1D.

- The text should include a note on the fact that IFN-gamma stimulation does not significantly affect phagocytosis (ie, the number of phagosomes formed is similar with/without IFN-gamma).

We have added that. IFNg does slight increase phagocytosis. But all data is normalised to protein amounts in blots and proteomics.

- Fig 1H: "UT" should be explained in the caption or text.

Done.

- The introduction of the different DUBs specificity lines 116-119 and Fig 1H lacks clarity and is sometimes inconsistent between text and figure. The meaning of the signs ">" and ">>" should be clearly stated. A suggestion would be to replace the top left box of Fig 1H with a table crossing the mentioned DUBs and their targets.

Changed text and figure

- Fig 1.I : A panel showing the full cell (like in Fig 3C) should be included.

We added a new fluorescence micrograph as requested.

- Fig 1.I: The scale bar is missing.

Added.

- Fig. 1I: Why beads of 3 microns of diameters are used here while the size used for phagosome isolation is 0.8 micron?

For microscopy purposes 3 um beads provide substantially better ability to resolve membrane and co-localisation. For proteomics, 0.8 um polystyrene beads are used as there is a better uptake and it reduces the overall number of cells (which is already very high!).

- The caption of Fig 1C-E should be untangled. It is currently hard to follow.

Done.

- In the paragraphs contained between lines 168-190, most references to Fig 2 subpanels are incorrect.

Done.

- Fig 2F is not described in the text.

Done.

- Supplementary Fig 2B is not described in the text.

Done.

- In Supplementary Fig 2, what do the blue and red parts of the SNARE sequences correspond to? This should appear in the caption. Besides TAB2 PD should be introduced in the caption (ie, "TAB2 PD is a K63-chain binding protein").

Done.

- Lines 220, 223, and Fig 3E, the term "upregulated" is incorrect and should be replaced by "enriched" as it refers to protein abundance on a subcellular compartment.

Done.

- Fig 3A: The results displayed are confusing in regard to the previous figures. My understanding is that the E3 ligases plotted in Fig 3A are selected from the list plotted in Fig 2C (this information, if correct, should appear somewhere in the text or caption, as well as the selection criteria). However, why Herc6 and RNF115 are written "IFN only" in Fig 2C - suggesting that they were below the detection level without IFN stimulation - but their log2 intensity in the unstimulated condition is display in Fig 3A?

As this was a label-free proteomics experiment, "IFN only" or "Resting only" mean that these E3 ligases were only detected in the respective samples and not the other. Therefore, a ratio is difficult to generate unless imputation of the detection level is used. Added following sentence into the figure legend: "IFN only" or "Rest only" are used when the proteins were only detected in the respective sample by label-free proteomics. we have made this clearer in the figure legend.

- Fig 3A: The carried t-tests show 4 stars in the comparison of RNF115 enrichment between Ctrl and IFN-gamma treatment but only 2 stars for Herc6 (which by eye look similar). To dissipate any misunderstanding, the authors may provide the raw data used for this figure in their rebuttal.

The data for this can be found in Supplementary Table 1. The p-values come from the imputation data using Perseus at default settings.

- Fig 3B: Rab7 cannot be used as a purity control as it interacts with RNF115 (as explained in lines 228-231) and should be removed from the plot.

We disagree with that. Rab7, which is very, very abundant on phagosomes (log2 intensity = 31), while RNF115 is low abundant as many regulatory proteins (log2 intensity = 20). While Rab7 does change to a degree, it still serves as a good marker of the endo-lysosomal compartment.

- Fig 3D: The proteins named within the volcano plot are not mentioned in the text or caption. It would be preferable to point at proteins that are later mentioned in the text (such as the one present in Fig 3F).

Some of the proteins in the volcano plot are now mentioned in the text.

- The results displayed in Supplementary Fig 3C-D are described lines 263-268 as such: "Ablation of RNF115 or loss of E2 binding of RNF115 did not affect phagocytic uptake of carboxylated particles (supplementary figure 3C) or bacterial uptake (supplementary figure 3D)." Yet, RNF115 mutant is only introduced in line 301, and the bacteria in line 327. I believe that the authors may have wrongly reshuffled the position of this line in their manuscript. This needs to be re-organized (see also additional suggestions).

We have now reorganised the manuscript.

- In Supplementary Fig 3: Does "RFU" stands for "relative fluorescence units" and "CytD" for "CytochalasinD"? This should appear in the caption. Similarly, the reason why CytochalasinD is used (I guess as a negative control ?) should be stated somewhere. The material and method section describes CFU determination but this does not appear in any figure. Maybe the authors meant "CFU" instead of "RFU" in the supplementary Fig 3D?

Done.

- Supplementary Fig 3D: The condition "RNF115 W259A" should be included.

Considering that we have shown multiple times that the W259A mutant behaves like a KO, we do not feel that this experiment is essential.

- Lines 278-279: the statement "loss of RNF115 also decreased recruitment of the ESCRT complex highlighting the role in ubiquitylation for the functional role of the complex" is not supported by the data because RNF115 is depleted but not replace by RNF115 W259A. Additional experiments should be performed or the statement should be corrected. Same comment for line 290 " this data shows that ubiquitylation plays an important role in protein and vesicle trafficking."

We have toned down the first sentence: "Moreover, loss of RNF115 also decreased recruitment of the ESCRT complex, highlighting a potential functional role for this complex in ubiquitylation. "
We believe our data, including new data in Figure 5 shows unequivocally that RNF115 and therefore its ubiquitylation function plays a role in protein and vesicle trafficking.

- Line 283, the authors wrote "TRAPP was significantly less abundant on RNF115 KO phagosomes and may explain the retention of cargo that was meant to be secreted". Was this retention of cargo observed? If yes, the authors should include such data or references to published work.

We cited literature about the TRAPP complex and its role in secretion. Our proteomics data shows that proteins with secretion signal are enriched in RNF115 KOs.

- Fig 4A: the result of IFN-gamma treatment is not commented in the text.

This has been added.

- Fig 5B: what is Bafilomycin used for? This and the interpretation of the results should appear in the text (or be removed from the figure).

Bafilomycin blocks acidification and thereby phagosome maturation. It serves as a negative control. We added this to the figure legend.

- Fig 5B: The conditions +/-IFN gamma treatment should be included.

This is in vivo data and therefore IFN-gamma treatment cannot be performed.

- Fig 5B: Strange lines appear around "WT+Bafi" and "RNF115+Bafi" lines instead of shaded area representing SEM.

Done.

- Lines 298-300: " These data were further validated by complementing RNF115 KO with RNF115-HA that reversed enhanced phagosomal proteolytic activity of KO macrophages". Where are the data?

We have re-organised the whole part and it should now be clearer.

- Lines 335-336: "The analysis revealed that RNF115 KO deficiency did not impact uptake of either pathogen" refers to results displayed in Supplementary Fig 4D, not Fig 4E. The following sentence "However, degradation of phagosomal S. aureus was significantly increased in the KO, while cytosolic Listeria were not affected" most likely refers to Fig 4E.

We have changed this part of the manuscript.

- Fig 4E: the "non-infected" condition must be shown to determine the gate of GFP-positive signal versus autofluorescence.

We have removed this figure.

- Fig 4E: the data doesn't allow to decipher if the lack of intracellular S. aureus is due to bacterial clearance or lack of replication. Several time points should be displayed to provide this information.

We have removed this. All infection experiments were tested at different times and the optimal time was used.

- Lines 366-367: the link between CD68-stained macrophage and tissue repair should be justified by literature references.

CD68 is a good marker for Kupffer cells in the liver. We have added a reference.

- Line 368: the conclusion on bacterial clearance is not supported by the data as the bacteria have not been quantified.

We have now added Salmonella data showing input/output data.

- Fig 5: Adding a condition with/without IFN-gamma stimulation would strengthen the results.

This is in vivo data and therefore no IFN-gamma can be added. While we would agree that an infection in RNF115 /IFN-gamma (or receptor) double KO mice would be very interesting, we feel it is out of scope for this study.

- In the material and methods, the bacteria are not described as fluorescent, but S. aureus GFP and L. monocytogenes eGFP are used in Fig 4E. The plasmid used should be mentioned somewhere.

We have added this information

- A description of the supplementary tables should be provided.

Done.

- Within the supplementary table containing the tabs "all proteins", "regulated", "unique IFNg", "Sheet4" and "Unique resting", the contains of the tabs don't match their title (ie, "unique IFNg" and "Unique resting" seem to have been exchanged).

We have changed that.

- Other points: Quotes in the introduction do not always fit the previous sentence (ie, line 45 "brown et al", line 54 "Jain et al"). The author should double-check that appropriate reference are used throughout the text.

Done.

- Additional non-essential suggestions for improving the study (which will be at the author's/editor's discretion):

- Depending on how the authors develop the next version of this manuscript, they may consider moving Fig 3D-F after Fig 4-5, as Fig 4-5 focus on the impact of RNF115 loss but do not explore the role of its potential downstream target.

We have performed substantially more experiments and hope that the reviewer will like them.

- The supplementary Fig 3 could be split into 2 supplementary figures, one including panels A-B and the other including panels C-D. The panels C-D could be moved later in the text.

Done.

- The authors have previously performed and published similar proteomic investigations, which are quoted in the text. They may clarify that all datasets displayed in this study have been freshly generated.

Yes, all data in this manuscript is freshly generated and has not been published before.

Referee #4 (Report for Author)

In this study, the authors investigate post-translation regulation of phagosomal maturation in macrophages by ubiquitylation of phagosomal-relevant vesicle trafficking proteins. Specifically, the authors demonstrate that i) phagosomal proteins in IFN-y-treated macrophages have increased ubiquitylation on specific lysine residues; ii) RNF115 is one such phagosomal-localized E3 ligase that negatively regulates phagosomal maturation through its ubiquitin ligase activity, and iii) regulation of phagosomal maturation by RNF115 has important repercussions in the inflammatory response against pathogenic bacteria such as, S.aureus. Currently in the literature, it is unclear as to how IFNγ treatment delays phagosome maturation in macrophages. However, the authors provide one explanation through ubiquitylation. Although ubiquitylation has been determined in previous studies to play a role in phagosome maturation, the authors have identified RNF115 as a specific E3 ligase that regulates this process and further characterized its role both in vitro and in vivo.

Overall, the data is novel, convincing, and high quality. The evidence provided adequately supports the authors major conclusions, with a few exceptions pertaining to the effect of ubiquitylation on phagosomal trafficking proteins and the direct ubiquitylation of phagosomal proteins by RNF115 (see major points). The greatest weakness of the study is that the authors demonstrate 'what' ubiquitylation does to phagosome maturation but very limitedly, 'how'. While the authors convincingly show RNF115's ubiquitin activity delays phagosome maturation in macrophages, it is unclear if this is due to the inability of relevant proteins to traffic to the phagosome (ie. protein recruitment is disrupted by ubiquitylation of proteins on the phagosomal membrane), phagosomes cannot acquire lysosomal proteins to mature (ie. ubiquitylation of phagosomal and lysosomal fusion proteins prevent phagosome-lysosome fusion due to disrupted protein interactions), both or through an alternative mechanism.

Major points:

1. Supplementary figure 2. This provides a rational argument as to how ubiquitylation negatively regulates phagosome maturation as phagosomes may not be able to fuse with lysosomes due to disrupted SNARE interactions. Firstly, I recommend moving this supplementary figure to the main paper. Secondly, it would strengthen the study to explore the effect of RNF115 KO on SNARE interactions through pulldown and/or colocalization etc. +/- INFg.

We have performed additional experiments showing that some of the SNAREs may be substrates of RNF115. The whole section around Figure 5 is new.

2. Can phagosomal fusion proteins localize to phagosomes due to ubiquitylation? In Figure 3F, I would like to see the proteomic data on lysosomal fusion proteins shown in Supplemental figure 2.

We have added substantial data around this in Figure 5.

3. Although the authors show that ubiquitylation of phagosomal proteins in increased in IFNγ-treated macrophages, RNF115 has not been demonstrated to directly ubiquitinate phagosomal proteins. To strengthen this claim, phagosomal ubiquitylation should be characterized in WT VS RNF115 KOs, such as Figure 1C, D, E, F and G.

Please see Figure 5.

Minor points:

1. Line 58. May be helpful to include a brief description of the roles of the E1, E2 and E3 ubiquitin enzymes.

Done.

2. Line 61. Missing K27. Clarify that methionine is the N-terminus. Better to rearrange the order of the residues and N terminus to match the text.

Done.

3. Figure 3F. I am not convinced that there is no significance between the WT and RNF115KO for proteins Ap3d1 and Chmpb1 yet almost every lysosomal protein has significance.

Ap3d1 and Chmpb1 are not significant. We have added the data nonetheless as they have a clear direction of change. Together with the information that these proteins form functional complexes, we believe this data is compelling to show that the overall complexes are changing and not just single proteins.

4. Figure 4. Why does 4B and 4C have shaded areas for SEM but not 4A? In 4B, it looks as though there are 4 lines mid-graph (ie. the lines representing WT+Bafi and RNF115 KO+Bafi). This should be fixed for clarity. Further, in 4A and 4C, it may be better to change "KO" in the graph legend to "RNF115 KO" like the rest of the graph legends in the figure for clarity.

The shaded areas are so small that they cannot be seen.

5. Line 289. Clarify that phagosomes fluoresce when the lumen has high proteolytic activity, not due to acidity like pHrodo.

Added.

6. Lines 523 and 527. Was DQ red BSA used or green (see line 289)?

Well spotted. It was DQ-red! We changed that.

7. Lines 630 and 650: TSB abbreviation used twice.
Done.

1st Revision - Editorial Decision — 30th Aug 2022

Thank you again for submitting your revised manuscript for our consideration. It has now been re-reviewed by the three of the original referees, whose comments are copied below. As you will see, while referees 2 and 4 are satisfied with the revisions and only have minor remaining comments, referee 1 still retains several more substantive reservations, related in part to the new data, but in many cases also to experimental or presentational issues they had already raised during the initial round of review.

Given the previous extensions of the study and the fact that you have already addressed a considerable number of concerns during the first revision, I would in this case be happy to grant an exceptional second round of revision to allow you to deal with these remaining presentational and conceptual points - and to provide a careful point-by-point response to the remaining comments by referee 1.

Referee #1:

Referee #1 - Comments on revised version of EMBOJ-2021-108970R, „The ubiquitin ligase RNF115..."

The authors have satisfactory replied to some comments and not to others (below). This is very good mass spectrometry-based analytic work and the inventory of ubiquitin-modified phagosome proteins will be helpful for many scientists who are interested in a particular phagosome protein and want to know more about its modifications. The infection part, in particular, is not as convincing.

Authors' response to comment 3 original review: The authors essentially ignored the comments by summarizing that different pathogens affect the proteasome different - a view I share. Therefore, they changed from one pathogen (Listeria) to another (Salmonella), keeping the previous Staphylococcus model. In how did that solve the problem or answer the question? They still use two intracellular pathogens, each of which has their pecularities, as the authors state themselves. Also, I would have expected that there would be some specific response to my original comments now that these comments had been made.

Authors' response to previous comment 4 original review: The authors write about high molecular weight signals in the Rab7 immunoblot, "we have highlighted that in the figure", yet they did not.

Authors' response to previous comment 14 original review indicating (in response to my question) that their original comment that SNARE proteins could be inhibited by ubiquitylation was speculation. Yet, the authors present the legend to Suppl Fig. 2 again in a way which implies that inhibition of SNARE activity by ubiquitylation was proven.

Revision comment 1: Fig. 1I and Fig. 3C: These are very nice micrographs but I really have a hard time seeing any colocalization when I define colocalization of phagosomes with a membrane marker molecules (auch as LAMP1) as "membrane proteins are present on the circumference of the phagosome at clearly higher concentration than in the phagosome's direct environment". This is indeed and clearly seen, e.g., in Fig. 5C, but not here.

Revision comment 2: Fig. 3 legend reads "RNF115 affects several phagosome functions". This is not shown here. The whole figure is about presence and absence not about function.

Revision comment 3: Fig 6A: What does "Output/Input" mean? This is explained neither in the figure legend nor in the Methods section Lanes 793-801). Are all added bacteria before gentamicin pulse an "Input" or all bacteria after gentamicin (which "Materials" seem to indicate)? Only 0.5% to 1% of bacteria survive the procedure. It would be hard to understand how 99.5% killing of intracellular bacteria versus 99.0% should be biologically meaningful, even if statistically significant. Even more so as Salmonella normally multiplies in BMDMs, even at early times which was not considered here.

Revision comment 4: The authors write that "the phagosome, as part of the endo-lysosomal system, interacts with practically all vesicle trafficking pathways in the cell and therefore allows identifying specific pathways that RNF115 may play a role in". First, I would not concur with this statement - do peroxisomes, COPI vesicles and mitochondrial OM vesicles all fuse with phagosomes possibly followed by retrieval? And: If everything intersects with the vacuole, is then the analysis of a specific role of an RNF protein not much harder than if it served in just one pathway to the lysosome? And following this, lanes 288-299: This is a partial listing of what the protein lists may mean. There is no biological explanation. What, e.g., does it potentially mean when peroxisomal proteins are "reduced" on a phagosome? What does it mean when rhe ESCRT complex is less reduced (or more released)?

Revision comment 5: Fig. 4C lacks the color code for "KO + RNF115". Standard deviation should be used instead of Standard Error of the Mean and is phagosomal acidification in (B) really increased if one considers the lower starting "0 min" point ("min" missing in this fig) for the WT sample? This effect, even if it were mathematically "significant" (with SD instead of SEM), might not be biologically significant at all. Yet see next point:

Revision comment 6: Line 452: "...we tested whether enhanced RNF115-mediated phagosome acidification impacts innate immunity...". I cannot see any part in Fig. 7 that would be presenting experimental manipulation or quantification of acidification.

Revision comment 7: Fig 5C. There seem to be problems with the general set-up of the experiment. The method of immunofluorescence does not have the resolution to judge whether two proteins are at the very same place, only in special set-ups like with FRET. If levels of proteins increase over the whole phagosome membrane, does that really provide evidence for a

functional relationship (between VAMP8 and syn7)? And l. 733: The authors state that phagosomes positive for both syn7 and VAMP8 were treated as regions of interest. Which proportion of all phagosomes did actually contain both the SNAREs? Was is different between WT and RNF115 KO cells?

Revision comment 8: Fig 6B: The implications of increased TNF-a / IL-6 for a Salmonella infection are not at all discussed. Would one expect more/less growth of bacteria with high TNF-a values? I suppose, TNF-a would limit multiplication, yet its production is stimulated in RNF115 KO (Fig. 6C) whereas multiplication is increased (Fig. 6A).

Revision comment 9: In the first round of review, I have commented on the presence of the gentamicin protection assay in the Materials and Methods section although the method was not used (any more?). Now, there is "L. monocytogenes" in the section (lane 796) although the bacteria have taken out of version 1. I am asking the authors to meticulously comb through their manuscript for old bits that are not meaningful (any more).

Revision comment 10: Fig. 2 B and C: Ratios are given between abundancies of proteins in 'IFN' and 'WT' samples: 'log2 IFN/WT'. Please change to 'log2 IFN-g/resting' as in Fig. 2 D.

Revision comment 11: l. 328: "This 'ligase dead' mutant increased proteolytic activity in macrophages, similar to the KO cells, demonstrating that RNF115 E3 ligase activity is needed for regulation of phagosome maturation (...)." Please rephrase: (1) The mutant increases proteolytic activity in macrophage phagosomes (not cells); (2) this is a rescue experiment in which WT or ligase-dead RNF115 were expressed in RNF115 KO (not WT) cells. The authors provide a better description and interpretation of these data in the caption of Fig. 4.

Revision comment 12: Fig. 6. The authors should briefly explain how the Salmonella reporters work. What does 50% "ON PssaG" mean? What kind of sample would be 0 or 100%? It is impossible for an uninitiated reader to evaluate the relevance of the small differences. Even the one indicated reference (Rollenhagen et a. 2004) did not present their data like this and gives no further insight into how the data wer calculated.

Revision comment 13: Fig. 7. The y-axis titles (A-E) should indicate what was measured rather than what the interpretation of the data is; e.g., Figure 7 A "hepatic lesion area [µm]" or similar instead of "% inflammation".

Referee #2:

The authors have received critical comments from four reviewers, and they have made a substantial effort to address them. This included the inclusion of novel experimental data and considerable revisions of the structure and concept of the manuscript. This has resulted in a great improvement of the quality of the paper.

Perhaps a remaining point to emphasize is that, as shown in Figure 2, the proteomics indicated that IFN-γ activation increases ubiquitylation of innate immunity and vesicle trafficking proteins. As one of the reviewers stated, there is some intersection with ISG15ylation (e.g. herc6, trim25, Figure 2B/C). The authors state that ubiquitylation is generally much more abundant in cells and ISG15ylation typically induced by type I interferon. However, there are many Lys residues that can be both, ubiquitylated and ISG15ylated, which are indistinguishable at the GlyGly-Lys level detected by shotgun proteomics, and levels of the latter can reach high levels at the peak of IFN induction. This should be acknowledged by the authors as one of the potential limitations of this study.

Other than that, this reviewer would recommend the revised version to be considered for publication.

Referee #4:

The revisions have addressed my major concerns. I suggest however that the results from the new figure 5 should be briefly summarized in the discussion e.g. before Line 564-565. E.g.: "Some molecular targets of RNF115 were identified to be membrane fusion machinery. These ubiquitylated targets may regulate phagosome maturation by preventing lysosomal contribution to maturing phagosomes.". The authors should be congratulated on their contribution to the field.

Rebuttal for "The E3 ubiquitin ligase RNF115 regulates phagosome maturation and host response to bacterial infection"

We would like to thank all reviewers for providing their valuable time and improving our manuscript. It is much appreciated.

Referee #1:

Referee #1 - Comments on revised version of EMBOJ-2021-108970R, „The ubiquitin ligase RNF115..."

The authors have satisfactory replied to some comments and not to others (below). This is very good mass spectrometry-based analytic work and the inventory of ubiquitin-modified phagosome proteins will be helpful for many scientists who are interested in a particular phagosome protein and want to know more about its modifications. The infection part, in particular, is not as convincing.

Authors' response to comment 3 original review: The authors essentially ignored the comments by summarizing that different pathogens affect the proteasome different - a view I share. Therefore, they changed from one pathogen (Listeria) to another (Salmonella), keeping the previous Staphylococcus model. In how did that solve the problem or answer the question? They still use two intracellular pathogens, each of which has their pecularities, as the authors state themselves. Also, I would have expected that there would be some specific response to my original comments now that these comments had been made.

To improve the answer to comment 3 of the original review, we have made several changes listed below:
1) The bacteria used in the flow cytometry assays express eGFP reporters, then trypan blue was used to quench any signals away from macrophages as previously described here in PMID: 6402550 and PMID: 27841303. But instead of using these cytometry data, we have used CFU analysis as it is traditionally used.
2) We have added the uptake data (time 0 h post infection) in Figure EV6A-B for *Salmonella* and *S. aureus* infections.
3) We have added the interferon-stimulated macrophages infections with *Salmonella* in Figure 6 and *S. aureus* in Figure 7A as well as in Figure EV6A-B for uptake (time 0 h post infection).
4) We have performed CFU analysis to quantify the number of bacteria at time 0 h post infection (p.i.) in Figure EV6 and 4h p.i. for *Salmonella* in Figure 6 and 6h p.i. for *S. aureus* in Figure 7.
5) We do not have a home office license the apathogenic *Listeria innocua* strain so we cannot infect cells or mice with this strain, but we have infected macrophages with *L. monocytogenes* (Figure 1).

Data using *L. monocytogenes*

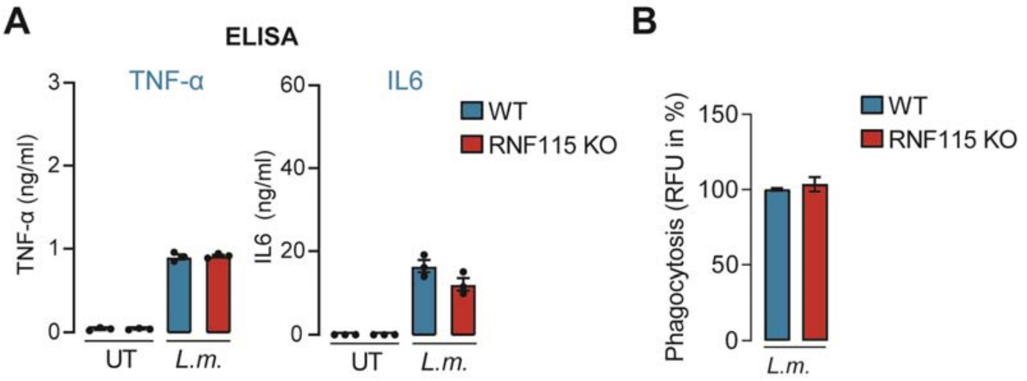

Figure 1. Data using *L. monocytogenes*. A) TNF-a and IL6 ELISA, **B)**. Phagocytosis assay. The data show that there are no differences with Listeria, most likely since it is escaping the phagosome early.

6.) We have removed *Listeria* altogether from the manuscript to avoid confusion for readers. We have switched to *Salmonella* as *Listeria* escapes the phagosome very early after phagocytosis while *Salmonella* remains in the phagosome longer making it a better intracellular pathogen to study the effect of phagosome maturation on bacterial survival.

Authors' response to previous comment 4 original review: The authors write about high molecular weight signals in the Rab7 immunoblot, "we have highlighted that in the figure", yet they did not.
We appreciate the reviewer for pointing this out as it was an error exporting the .tif file. We have highlighted the ubiquitylation of Rab7 in Figure EV3B.

Authors' response to previous comment 14 original review indicating (in response to my question) that their original comment that SNARE proteins could be inhibited by ubiquitylation was speculation. Yet, the authors present the legend to Suppl Fig. 2 again in a way which implies that inhibition of SNARE activity by ubiquitylation was proven.
We have changed the original legend of EV3A '*Ubiquitylation sites of phagosomal SNARE proteins are almost entirely within SNARE domains, thereby blocking SNARE protein interactions.*' with '*Ubiquitylation sites of phagosomal SNARE proteins are almost entirely within SNARE domains, thereby this might block SNARE protein interactions.*'

Revision comment 1: Fig. 1I and Fig. 3C: These are very nice micrographs but I really have a hard time seeing any colocalization when I define colocalization of phagosomes with a membrane marker molecules (auch as LAMP1) as "membrane proteins are present on the circumference of the phagosome at clearly higher concentration than in the phagosome's direct environment". This is indeed and clearly seen, e.g., in Fig. 5C, but not here.

We have remade Fig. 1I to contain a slices view of a bead-induced phagosome stained with K63-linkage specific polyubiquitin as well as the membrane marker Rab7a. we tried to focus on a very tight z-stack.

With regards to the RNF115 and LAMP1 staining, the reviewer makes a very good point. We did not mean to say that these two proteins are colocalizing. We only wanted to show that RNF115 is associated with LAMP1-staining phagosomes. We have changed the sentence to read **"Consistent with immunoblot data, immunofluorescence microscopy analysis revealed RNF115 association with phagosomes by partly co-staining with lysosomal-associated membrane protein 1, LAMP1, in IFN-γ activated macrophages"**. We have also replaced colocalization with co-staining in the figure legend.

To account for people with red-green colour-blindness, we have represented Figure 1I and Figure 3C in a magenta/cyan/yellow version in Figure EV2 and Figure EV5, respectively.

Revision comment 2: Fig. 3 legend reads "RNF115 affects several phagosome functions". This is not shown here. The whole figure is about presence and absence not about function.

We have modified Figure 3 title as 'Ubiquitin E3 ligase RNF115 is enriched on phagosomes of IFN-γ activated macrophages and loss of RNF115 affects several phagosomal protein compositions'

Revision comment 3: Fig 6A: What does "Output/Input" mean? This is explained neither in the figure legend nor in the Methods section Lanes 793-801). Are all added bacteria before gentamicin pulse an "Input" or all bacteria after gentamicin (which "Materials" seem to indicate)? Only 0.5% to 1% of bacteria survive the procedure. It would be hard to understand how 99.5% killing of intracellular bacteria versus 99.0% should be biologically meaningful, even if statistically significant. Even more so as Salmonella normally multiplies in BMDMs, even at early times which was not considered here.

Output/input values correspond to the number of bacteria inside the cell recovered at the specific time point (output, CFU analysis) normalised over the number of bacteria added to the cells at the start of the infection (multiplicity of infection, MOI). We have added this information in the caption of Figure 6A and in Material and Methods.

In order to clarify any confusion about the bacterial infection experiments we have improved this protocol in the Material and Methods: 'All bacteria were used at mid-exponential phase for infection experiments, washed in ice cold PBS twice and, subsequently, re-suspended in cell culture media at the desired MOI (10 for S. Typhimurium and 25 for S. aureus). After 30 min (uptake, time 0), cells were washed and incubated with fresh media containing 50 μg/ml gentamycin for 1 h and 15 μg/ml thereafter (S. Typhimurium) or 23 μg/ml for 30 min and 0.23 μg/ml gentamycin thereafter (S. aureus). Colony forming units (CFUs) were determined by lysing cells at indicated time points in 0.1% Triton-X 100 in PBS, from which serial dilutions were plated for overnight incubation at 37 ˚C. The following day bacterial colonies were counted and CFU was determined. We calculated the output/input values correspond to the number of bacteria inside the cell recovered at the specific time point (output, CFU analysis) normalised over the number of bacteria added to the cells at the start of the infection.'

We agree with the reviewer. Salmonella multiplies in some macrophages in *in vivo* experiments in mice. However, replication time for Salmonella *in vivo* has been estimated to be 6 hours. Our infections are at 4 h and in *in vitro* experiments in BMDM cells. In order to clarify these data, we have added the percentages of uptake in BMA and BMDM cells after *Salmonella* and *S. aureus* infection in Figure EVA-B.

Revision comment 4: The authors write that "the phagosome, as part of the endo-lysosomal system, interacts with practically all vesicle trafficking pathways in the cell and therefore allows identifying specific pathways that RNF115 may play a role in". First, I would not concur with this statement - do peroxisomes, COPI vesicles and mitochondrial OM vesicles all fuse with phagosomes possibly followed by retrieval? And: If everything intersects with the vacuole, is then the analysis of a specific role of an RNF protein not much harder than if it served in just one pathway to the lysosome? And following this, lanes 288-299: This is a partial listing of what the protein lists may mean. There is no biological explanation. What, e.g., does it potentially mean when peroxisomal proteins are "reduced" on a phagosome? What does it mean when rhe ESCRT complex is less reduced (or more released)?

After 17 years of phagosome proteomics experience, we can state quite clearly that the phagosome indeed interacts with almost all vesicle trafficking pathways (PMID: 31398943, PMID: 28432021, PMID: 26494048).We think this may due to the stochastic nature of vesicle trafficking in which vesicles fuse and then – if not in the right location – are retrieved again and "sent on their way". We see indeed proteins involved in COPI vesicles as well as some mitochondrial proteins.
Interestingly, only some of the mitochondrial proteins are present, suggesting that they may be deriving from mitochondrial vesicles. We have toned down this state to *"the phagosome, as part of the endo-lysosomal system, interacts with* ***many*** *vesicle trafficking pathways in the cell".*

The reviewer is right that the data show indeed that the role of RNF115 may be multiple and that the overall biology is complicated. More research, probably mostly in the cell biology field, will be required to identify more roles for RNF115. We feel that we have defined a novel and exciting role for RNF115 in phagosome biology and we hope that others may pick up from here.

It is difficult to say what the effects of reduced ESCRT or peroxisomal proteins on the phagosome are. We believe one part of the power of proteomics is that we can highlight interesting observations which can then be followed up by experts in these fields. Unfortunately, a further analysis of these effects would be out of scope for this manuscript.

Revision comment 5: Fig. 4C lacks the color code for "KO + RNF115". Standard deviation should be used instead of Standard Error of the Mean and is phagosomal acidification in (B) really increased if one considers the lower starting "0 min" point ("min" missing in this fig) for the WT sample? This effect, even if it were mathematically "significant" (with SD instead of SEM), might not be biologically significant at all. Yet see next point:
We appreciate the reviewer for pointing this out as it was an error exporting the .tif file. In addition, we have improved the figure 4: 'KO' is labelled as 'RNF115 KO' and 'Bafi' as 'Bafilomycin A1'. Changes in phagosome acidification represent a minor fold-change compared to changes in proteolysis (PMID: 31400233). Moreover, Figure 5D has the same colour code than other figures (blue for WT and red for RNF115 KO).

Revision comment 6: Line 452: "...we tested whether enhanced RNF115-mediated phagosome acidification impacts innate immunity...". I cannot see any part in Fig. 7 that would be presenting experimental manipulation or quantification of acidification.
Indeed acidification specifically with *S. aureus* has not been tested just with beads thus sentence has been modified to: "…we tested whether RNF115-mediated phagosome modifications impacts innate immune sensing…"

Revision comment 7: Fig 5C. There seem to be problems with the general set-up of the experiment. The method of immunofluorescence does not have the resolution to

judge whether two proteins are at the very same place, only in special set-ups like with FRET. If levels of proteins increase over the whole phagosome membrane, does that really provide evidence for a functional relationship (between VAMP8 and syn7)? And l. 733: The authors state that phagosomes positive for both syn7 and VAMP8 were treated as regions of interest. Which proportion of all phagosomes did actually contain both the SNAREs? Was is different between WT and RNF115 KO cells?
While we acknowledge that FRET is indeed the gold standard for examining protein localisation and interaction, this was not the intended aim of the experiment as it is already well-known that Syntaxin 7 and VAMP8 interact with one another and are part of a multiprotein complex that is involved in endosomal fusion (**see for example PMID: 11101518 & PMID: 15363411**). We simply wanted to show that these two proteins exist in the same general area on the phagosome. We would also like to draw the reviewer's attention to this paper that describes the resolution of Airyscan down to 120 nm (see: https://www.nature.com/articles/nmeth.f.404#article-info), which should be able to resolve a large protein complex, for example. **We have however, modified our text to be more in line with your comments and changed "colocalization" to "colocation", as well as modifying the sentence prior to read "Fluorescence microscopy validated a relatively high level of colocation between VAMP8 and STX7 around the phagosome membrane" and "Loss of RNF115 increased the colocation slightly, but significantly, indicating that RNF115-induced ubiquitylation of this complex may regulate its trafficking or complex formation"**. We have also recoloured all microscopy images to use a cyan/magenta/yellow format to account for readers who are red-green colour blind in Figure EV2 and Figure EV5.

Regarding the proportion of phagosomes that were SNARE-positive, we originally quantified these events and found there to be ~90 SNARE-positive phagosomes across 13 fields of view, with multiple SNARE-positive phagosomes observable within a single field of view. Considering that not all beads in a typical field of view will be phagocytosed, we consider this to be biologically significant. In terms of quantifying this between WT and RNF115 KO cells we found that while WT cells tended to have more SNARE-positive phagosomes (based on Syntaxin7 staining), this was not statistically significant. Additionally, due to SNARE staining not being confined to the phagosome, as well as the dynamic intensities around phagosomes themselves, making a true quantification would be incredibly difficult without introducing some bias into the analysis. For the reviewers' interest, we have included a representative image below demonstrating the abundance of SNARE-positive phagosomes (indicated by arrows) as well as a comparison of these events between WT and RNF115 KO cells below this. The two panels at the top are from WT cells, where the red represents Syntaxin 7 staining, and the white are the silica beads in the right panel.

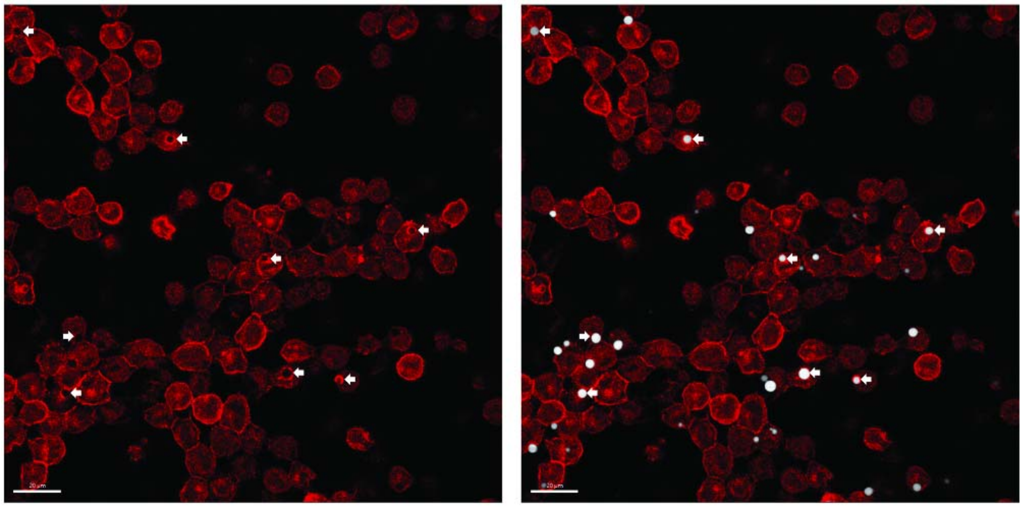

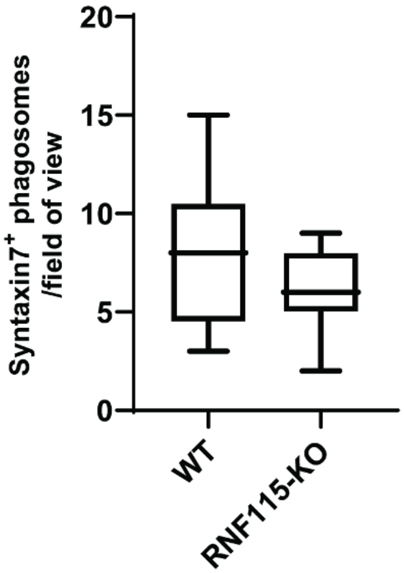

Revision comment 8: Fig 6B: The implications of increased TNF-a / IL-6 for a Salmonella infection are not at all discussed. Would one expect more/less growth of bacteria with high TNF-a values? I suppose, TNF-a would limit multiplication, yet its production is stimulated in RNF115 KO (Fig. 6C) whereas multiplication is increased (Fig. 6A).
Induction of TNF-α secretion can already be detected at 6h post-infection with *Salmonella* but for this to have an actual effect on bacterial killing the time-point is too early. To see how far TNF-α would affect bacterial killing in RNF115 KO later time points should have been tested. In our hands at later time points the cells were very stressed and started dying, thus no reliable conclusions could be made from later time points.

Revision comment 9: In the first round of review, I have commented on the presence of the gentamicin protection assay in the Materials and Methods section although the method was not used (any more?). Now, there is "L. monocytogenes" in the section (lane 796) although the bacteria have taken out of version 1. I am asking the authors to

meticulously comb through their manuscript for old bits that are not meaningful (any more).
We have removed any reference to the *Listeria* experiments/data from the manuscript.

Revision comment 10: Fig. 2 B and C: Ratios are given between abundancies of proteins in 'IFN' and 'WT' samples: 'log2 IFN/WT'. Please change to 'log2 IFN-g/resting' as in Fig. 2 D.
We have labelled Figure 2B and C as 'log2 IFN-γ/resting'. In addition, we have labelled Figure 3A as 'Resting' and Figure 3D as 'log2 (RNF115 KO/WT)'.

Revision comment 11: l. 328: "This 'ligase dead' mutant increased proteolytic activity in macrophages, similar to the KO cells, demonstrating that RNF115 E3 ligase activity is needed for regulation of phagosome maturation (...)." Please rephrase: (1) The mutant increases proteolytic activity in macrophage phagosomes (not cells); (2) this is a rescue experiment in which WT or ligase-dead RNF115 were expressed in RNF115 KO (not WT) cells. The authors provide a better description and interpretation of these data in the caption of Fig. 4.
The text has been changed to better explain the rescue experiment in RNF115 KO using WT and mutated version of the E3 ligase.

Revision comment 12: Fig. 6. The authors should briefly explain how the Salmonella reporters work. What does 50% "ON PssaG" mean? What kind of sample would be 0 or 100%? It is impossible for an uninitiated reader to evaluate the relevance of the small differences. Even the one indicated reference (Rollenhagen et a. 2004) did not present their data like this and gives no further insight into how the data wer calculated.
We have changed '% ON PsseG' and 'ON Pasr' by '% GFP', and this sentence was added to the figure caption: 'Percentage (%) of GFP was analysed from Salmonella-infected BMA cells for 4 h. C) % GFP of *Salmonella* encoding SPI-2 response (PsseG)-inducible promoter fused to GFP and D) acidity exposure (Pasr)-inducible promoter fused to GFP. Stronger SPI-2 response (PsseG) and acidity exposure (Pasr) was shown in RNF115 KO compared to WT cells.'

This is a functional assay for promoters ('…Salmonella reporter strains encoding stress-inducible promoters fused to an unstable GFP variant for acidity exposure (Pasr) and SPI-2 response (PsseG) were kindly provided by Dirk Bumann (Biozentrum Basel'), in this case *Salmonella* reporter strains encoded stress-inducible promoters fused to an unstable GFP variant for acidity exposure (Pasr) and SPI-2 response (PsseG). When these promoters are not activated, their GFP signal is 0%, no bacteria induce GFP expression. However, when the promoters are induced by the appropriate factors, the bacteria express GFP. If all the bacteria analysed by flow cytometry express GFP, we will have a 100 % signal.

Revision comment 13: Fig. 7. The y-axis titles (A-E) should indicate what was measured rather than what the interpretation of the data is; e.g., Figure 7 A "hepatic lesion area [µm]" or similar instead of "% inflammation".

We have labelled the y-axis title for Figure 7C as '% Hepatic lesion area', Figure 7D as 'Number of $CD3^+$ T-cell' and Figure 7E as 'Number of $CD68^+$ macrophages'.

Referee #2:

The authors have received critical comments from four reviewers, and they have made a substantial effort to address them. This included the inclusion of novel experimental data and considerable revisions of the structure and concept of the manuscript. This has resulted in a great improvement of the quality of the paper.

Perhaps a remaining point to emphasize is that, as shown in Figure 2, the proteomics indicated that IFN-γ activation increases ubiquitylation of innate immunity and vesicle trafficking proteins. As one of the reviewers stated, there is some intersection with ISG15ylation (e.g. herc6, trim25, Figure 2B/C). The authors state that ubiquitylation is generally much more abundant in cells and ISG15ylation typically induced by type I interferon. However, there are many Lys residues that can be both, ubiquitylated and ISG15ylated, which are indistinguishable at the GlyGly-Lys level detected by shotgun proteomics, and levels of the latter can reach high levels at the peak of IFN induction. This should be acknowledged by the authors as one of the potential limitations of this study.
The uncertainty of how far ubiquitin sites might be ISG15 sites have been acknowledged.

Other than that, this reviewer would recommend the revised version to be considered for publication.

Referee #4:

The revisions have addressed my major concerns. I suggest however that the results from the new figure 5 should be briefly summarized in the discussion e.g. before Line 564-565. E.g.: "Some molecular targets of RNF115 were identified to be membrane fusion machinery. These ubiquitylated targets may regulate phagosome maturation by preventing lysosomal contribution to maturing phagosomes.". The authors should be congratulated on their contribution to the field.

The summary sentence of the SNARE complex has been added to the discussion.

2nd Revision - Editorial Decision 6th Oct 2022

Thank you for submitting your final revised manuscript for our consideration. I have now looked through your responses to the last round of review, and I am pleased to inform you that I have now decided to accept the study for publication in The EMBO Journal.

EMBO Press Author Checklist

Corresponding Author Name: Matthias Trost	
Journal Submitted to: EMBO Journal	
Manuscript Number: EMBOJ-2021-108970R	

USEFUL LINKS FOR COMPLETING THIS FORM
The EMBO Journal - Author Guidelines
EMBO Reports - Author Guidelines
Molecular Systems Biology - Author Guidelines
EMBO Molecular Medicine - Author Guidelines

Reporting Checklist for Life Science Articles (updated January
This checklist is adapted from Materials Design Analysis Reporting (MDAR) Checklist for Authors. MDAR establishes a minimum set of requirements in transparent reporting in the life sciences (see Statement of Task: 10.31222/osf.io/9sm4x). Please follow the journal's guidelines in preparing your
Please note that a copy of this checklist will be published alongside your article.

Abridged guidelines for figures
1. Data
The data shown in figures should satisfy the following conditions:
- the data were obtained and processed according to the field's best practice and are presented to reflect the results of the experiments in an accurate and unbiased manner.
- ideally, figure panels should include only measurements that are directly comparable to each other and obtained with the same assay.
- plots include clearly labeled error bars for independent experiments and sample sizes. Unless justified, error bars should not be shown for technical
- if n<5, the individual data points from each experiment should be plotted. Any statistical test employed should be justified.
- Source Data should be included to report the data underlying figures according to the guidelines set out in the authorship guidelines on Data

2. Captions
Each figure caption should contain the following information, for each panel where they are relevant:
- a specification of the experimental system investigated (eg cell line, species name).
- the assay(s) and method(s) used to carry out the reported observations and measurements.
- an explicit mention of the biological and chemical entity(ies) that are being measured.
- an explicit mention of the biological and chemical entity(ies) that are altered/varied/perturbed in a controlled manner.
- the exact sample size (n) for each experimental group/condition, given as a number, not a range;
- a description of the sample collection allowing the reader to understand whether the samples represent technical or biological replicates (including how many animals, litters, cultures, etc.).
- a statement of how many times the experiment shown was independently replicated in the laboratory.
- definitions of statistical methods and measures:
  - common tests, such as t-test (please specify whether paired vs. unpaired), simple χ2 tests, Wilcoxon and Mann-Whitney tests, can be unambiguously identified by name only, but more complex techniques should be described in the methods section;
  - are tests one-sided or two-sided?
  - are there adjustments for multiple comparisons?
  - exact statistical test results, e.g., P values = x but not P values < x;
  - definition of 'center values' as median or average;
  - definition of error bars as s.d. or s.e.m.

Please complete ALL of the questions below.
Select "Not Applicable" only when the requested information is not relevant for your study.

Materials

Newly Created Materials	Information included in the manuscript?	In which section is the information available? (Reagents and Tools Table, Materials and Methods, Figures, Data Availability Section)
New materials and reagents need to be available; do any restrictions apply?	Yes	Material and Methods

Antibodies	Information included in the manuscript?	In which section is the information available? (Reagents and Tools Table, Materials and Methods, Figures, Data Availability Section)
For **antibodies** provide the following information:<br>- Commercial antibodies: RRID (if possible) or supplier name, catalogue number and or/clone number<br>- Non-commercial: RRID or citation	Yes	Material and Methods

DNA and RNA sequences	Information included in the manuscript?	In which section is the information available? (Reagents and Tools Table, Materials and Methods, Figures, Data Availability Section)
**Short novel DNA or RNA including primers, probes**: provide the sequences.	Yes	Material and Methods

Cell materials	Information included in the manuscript?	In which section is the information available? (Reagents and Tools Table, Materials and Methods, Figures, Data Availability Section)
**Cell lines:** Provide species information, strain. Provide accession number in repository **OR** supplier name, catalog number, clone number, and/**OR** RRID.	Yes	Material and Methods
**Primary cultures:** Provide species, strain, sex of origin, genetic modification status.	Yes	Material and Methods
Report if the cell lines were recently **authenticated** (e.g., by STR profiling) and tested for mycoplasma contamination.	Yes	Material and Methods

Experimental animals	Information included in the manuscript?	In which section is the information available? (Reagents and Tools Table, Materials and Methods, Figures, Data Availability Section)
**Laboratory animals or Model organisms:** Provide species, strain, sex, age, genetic modification status. Provide accession number in repository **OR** supplier name, catalog number, clone number, **OR** RRID.	Yes	Material and Methods
**Animal observed in or captured from the field:** Provide species, sex, and age where possible.	Not Applicable	
Please detail **housing and husbandry conditions**.	Yes	Material and Methods

Plants and microbes	Information included in the manuscript?	In which section is the information available? (Reagents and Tools Table, Materials and Methods, Figures, Data Availability Section)
**Plants:** provide species and strain, ecotype and cultivar where relevant, unique accession number if available, and source (including location for collected wild specimens).	Not Applicable	
**Microbes:** provide species and strain, unique accession number if available, and source.	Yes	Material and Methods

Human research participants	Information included in the manuscript?	In which section is the information available? (Reagents and Tools Table, Materials and Methods, Figures, Data Availability Section)
If collected and within the bounds of privacy constraints report on age, sex and gender or ethnicity for all study participants.	Not Applicable	

Core facilities	Information included in the manuscript?	In which section is the information available? (Reagents and Tools Table, Materials and Methods, Figures, Data Availability Section)
If your work benefited from core facilities, was their service mentioned in the acknowledgments section?	Yes	Acknowledgements

Design

Study protocol	Information included in the manuscript?	In which section is the information available? (Reagents and Tools Table, Materials and Methods, Figures, Data Availability Section)
If study protocol has been **pre-registered, provide DOI in the manuscript**. For clinical trials, provide the trial registration number **OR** cite DOI.	Not Applicable	
Report the **clinical trial registration number** (at ClinicalTrials.gov or equivalent), where applicable.	Not Applicable	

Laboratory protocol	Information included in the manuscript?	In which section is the information available? (Reagents and Tools Table, Materials and Methods, Figures, Data Availability Section)
Provide DOI OR other citation details if **external detailed step-by-step protocols** are available.	Not Applicable	

Experimental study design and statistics	Information included in the manuscript?	In which section is the information available? (Reagents and Tools Table, Materials and Methods, Figures, Data Availability Section)
Include a statement about **sample size** estimate even if no statistical methods were used.	Yes	Material and Methods
Were any steps taken to minimize the effects of subjective bias when allocating animals/samples to treatment (e.g. **randomization procedure**)? If yes, have they been described?	Yes	Material and Methods
Include a statement about **blinding** even if no blinding was done.	Yes	Material and Methods
Describe **inclusion/exclusion criteria** if samples or animals were excluded from the analysis. Were the criteria pre-established?<br><br>If sample or data points were omitted from analysis, report if this was due to attrition or intentional exclusion and provide justification.	Not Applicable	
For every figure, are **statistical tests** justified as appropriate? Do the data meet the assumptions of the tests (e.g., normal distribution)? Describe any methods used to assess it. Is there an estimate of variation within each group of data? Is the variance similar between the groups that are being statistically compared?	Yes	Figure legends and Material and Methods

Sample definition and in-laboratory replication	Information included in the manuscript?	In which section is the information available? (Reagents and Tools Table, Materials and Methods, Figures, Data Availability Section)
In the figure legends: state number of times the experiment was **replicated** in laboratory.	Yes	Figure Legends
In the figure legends: define whether data describe **technical or biological replicates**.	Yes	Figure Legends

Ethics

Ethics	Information included in the manuscript?	In which section is the information available? (Reagents and Tools Table, Materials and Methods, Figures, Data Availability Section)
Studies involving **human participants**: State details of **authority granting ethics approval** (IRB or equivalent committee(s), provide reference number for approval.	Not Applicable	
Studies involving **human participants**: Include a statement confirming that **informed consent** was obtained from all subjects and that the experiments conformed to the principles set out in the WMA Declaration of Helsinki and the Department of Health and Human Services Belmont Report.	Not Applicable	
Studies involving **human participants:** For publication of **patient photos**, include a statement confirming that consent to publish was obtained.	Not Applicable	
Studies involving experimental **animals**: State details of **authority granting ethics approva**l (IRB or equivalent committee(s), provide reference number for approval. Include a statement of compliance with ethical regulations.	Yes	Material and Methods
Studies involving **specimen and field samples:** State if relevant **permits** obtained, provide details of authority approving study; if none were required, explain why.	Not Applicable	

Dual Use Research of Concern (DURC)	Information included in the manuscript?	In which section is the information available? (Reagents and Tools Table, Materials and Methods, Figures, Data Availability Section)
Could your study fall under dual use research restrictions? Please check biosecurity documents and list of **select agents and toxins** (CDC): https://www.selectagents.gov/sat/list.htm	Not Applicable	
If you used a select agent, is the security level of the lab appropriate and reported in the manuscript?	Not Applicable	
If a study is subject to dual use research of concern regulations, is the name of the **authority granting approval and reference number** for the regulatory approval provided in the manuscript?	Not Applicable	

Reporting

The MDAR framework recommends adoption of discipline-specific guidelines, established and endorsed through community initiatives. Journals have their own policy about requiring specific guidelines and recommendations to complement MDAR.

Adherence to community standards	Information included in the manuscript?	In which section is the information available? (Reagents and Tools Table, Materials and Methods, Figures, Data Availability Section)
State if relevant guidelines or checklists (e.g., **ICMJE, MIBBI, ARRIVE, PRISMA**) have been followed or provided.	Not Applicable	
For **tumor marker prognostic studies,** we recommend that you follow the **REMARK** reporting guidelines (see link list at top right). See author guidelines, under 'Reporting Guidelines'. Please confirm you have followed these guidelines.	Not Applicable	
For **phase II and III randomized controlled trials**, please refer to the **CONSORT** flow diagram (see link list at top right) and submit the CONSORT checklist (see link list at top right) with your submission. See author guidelines, under 'Reporting Guidelines'. Please confirm you have submitted this list.	Not Applicable	

Data Availability

Data availability	Information included in the manuscript?	In which section is the information available? (Reagents and Tools Table, Materials and Methods, Figures, Data Availability Section)
Have **primary datasets** been deposited according to the journal's guidelines (see 'Data Deposition' section) and the respective accession numbers provided in the Data Availability Section?	Yes	
Were **human clinical and genomic datasets** deposited in a public access-controlled repository in accordance to ethical obligations to the patients and to the applicable consent agreement?	Not Applicable	
Are **computational models** that are central and integral to a study available without restrictions in a machine-readable form? Were the relevant accession numbers or links provided?	Not Applicable	
If publicly available data were reused, provide the respective **data citations in the reference list**.	Not Applicable	
